# Effects of integrating self-efficacy strategies into multi-joint resistance exercise for overweight and obese older adults: A randomized controlled trial

**DOI:** 10.1371/journal.pone.0335830

**Published:** 2026-08-21

**Authors:** Oratai Kanlayawut, Raweewan Maphong, Waris Wongpipit, Thanomwong Kritpet

**Affiliations:** 1 Faculty of Sports Science, Chulalongkorn University, Bangkok, Thailand; 2 Division of Health and Physical Education, Faculty of Education, Chulalongkorn University, Bangkok, Thailand; 3 Department of Sports Science and Physical Education, Faculty of Education, The Chinese University of Hong Kong, Hong Kong People’s Republic of China; 4 Center of Excellence in Educational Invention and Innovation, Faculty of Education, Chulalongkorn University, Bangkok, Thailand; ISSEP Kef: Universite de Jendouba Institut Superieur du Sport et de l’Education Physique du Kef, TUNISIA

## Abstract

Obesity among older adults is increasing globally and contributes to functional decline, highlighting the need for interventions that address both physical and psychological barriers to exercise. However, it remains unclear whether embedding self-efficacy strategies within each session of progressive multi-joint resistance training provides added benefits beyond resistance training alone. This study examined the additional effects of integrated self-efficacy strategies on body composition (primary outcome) and secondary outcomes including blood biomarkers, physical function, and psychological measures in overweight and obese older adults. Thirty-four adults aged 60–75 years were included in the final intention-to-treat analysis and randomly assigned to a multi-joint exercise group (MJ; n = 16; age 67.63 ± 5.28 years; mean ± SD) or a multi-joint exercise plus self-efficacy group (MJS; n = 18; age 67.35 ± 3.69 years). Both groups completed a 12-week progressive resistance training program (60 min/session, three times/week). Outcomes were assessed pre- and post-intervention. Data were analyzed using linear mixed-effects models (intention-to-treat), testing group × time interactions with estimated marginal means and 95% confidence intervals (CIs). Both groups improved physical function (muscle strength, endurance, balance, and mobility), with no significant group × time interactions. For resting heart rate, the MJS group showed minimal change (Δ = 0.8 beats·min ⁻ ^1^), whereas the MJ group increased (Δ = 7.8 beats·min ⁻ ^1^), yielding a between-group difference in change of −7.0 beats·min ⁻ ^1^ (95% CI −12.5 to −1.9; p = 0.009). A significant group × time interaction was observed for HbA1c (p = 0.014); however, Bonferroni-adjusted pairwise comparisons did not demonstrate statistically significant within-group changes. Psychological outcomes improved more in MJS, including knowledge, self-efficacy, and outcome expectations. In conclusion, multi-joint resistance training improves physical function in overweight and obese older adults. Embedding self-efficacy strategies within training may provide additional psychological benefits and selected physiological benefits beyond exercise alone.

## Introduction

The global rise in overweight and obese older adults presents an increasing challenge for healthcare systems. According to the World Health Organization, the age-standardized obesity rate among adults aged 18 years and older increased from 6.6% in 1990 to 15.8% in 2022 [1]. Excess weight in later life contributes substantially to functional decline, loss of independence, and increased premature mortality [2]. Consequently, effective interventions that promote healthy aging must address not only physiological limitations but also the psychological barriers that hinder regular participation in physical activity [3].

Among these barriers, physical discomfort is a prominent factor discouraging older adults from engaging in exercise. Such discomfort is frequently associated with reduced exercise self-efficacy, defined as an individual’s belief in their capability to perform physical activity, which in turn limits participation and exacerbates adverse health outcomes [4–6]. Bandura’s self-efficacy theory identifies perceived self-capability and expected outcomes as central determinants of behavior change. Interventions grounded in this theory, employing strategies such as mastery experiences, social modeling, and verbal encouragement, have been shown to enhance confidence and support sustained behavioral change [7]. Consistent evidence indicates that self-efficacy–based strategies can promote physical activity among overweight and obese older adults, leading to greater energy expenditure, improved exercise adherence, and more favorable weight-related outcomes [8,9].

Several intervention studies in older adults have therefore examined the combination of structured exercise programs with psychological or behavioral components, particularly those rooted in self-efficacy theory. These combined interventions have generally reported improvements in exercise adherence, motivation, and perceived competence, with some studies also demonstrating favorable effects on cardiometabolic indicators and functional performance [10,11]. Commonly applied psychological strategies include goal setting, feedback, self-monitoring, and social support, which aim to strengthen confidence and reduce perceived barriers to physical activity [12,13]. Nevertheless, the magnitude and consistency of physiological and functional benefits beyond exercise alone have varied across studies, suggesting that the additive effects of psychological components remain inconclusive.

Importantly, many previous studies have delivered psychological components as brief educational sessions or adjunct counseling conducted separately from exercise training [14], rather than systematically integrating self-efficacy strategies into each exercise session. In addition, much of the existing literature has focused on aerobic or mixed exercise modalities [15], with relatively limited emphasis on resistance-based, multi-joint exercise programs that more closely reflect the functional demands of daily living in older adults. As a result, evidence remains limited regarding whether embedding self-efficacy strategies throughout progressive resistance training can yield additional benefits for physical function and metabolic health in overweight and obese older adults.

While psychological readiness is an important determinant of exercise participation, it must be accompanied by appropriate physical training to elicit meaningful health improvements. Resistance training, particularly multi-joint exercises involving large muscle groups and functional movements such as squatting and stair climbing, has been shown to improve muscle strength, body composition, and aerobic capacity in older adults [16]. These exercises closely resemble activities of daily living and often provide greater functional benefits than single-joint movements. Even when training volume is matched, multi-joint resistance exercises have demonstrated superior gains in muscle strength and cardiovascular fitness [17]. Despite these advantages, adherence to resistance training recommendations remains suboptimal among overweight and obese older adults, frequently due to persistently low self-efficacy [18].

However, to date, randomized evidence remains limited regarding whether systematically embedding self-efficacy strategies within each session of progressive multi-joint resistance training confers additional physiological and functional benefits beyond resistance exercise alone in overweight and obese older adults. This persistent gap highlights the need for interventions that simultaneously address the psychological and physical dimensions of behavior change. Although self-efficacy enhancement and resistance training independently confer health benefits, few studies have integrated these elements into a single, theory-driven intervention. Importantly, embedding self-efficacy strategies directly within exercise sessions may influence not only participation but also the quality of engagement with training, including effort regulation, movement confidence, and tolerance to physical discomfort. In this sense, self-efficacy may act as a behavioral and psychophysiological moderator that shapes how individuals respond to resistance training stimuli, rather than merely determining whether they participate. However, empirical evidence testing this mechanism within resistance-based interventions remains limited. By comparing this integrated approach with a standard resistance training program lacking psychological components, this study seeks to clarify the added value of embedding self-efficacy principles within resistance exercise. The findings may inform the development of scalable, community-based programs that promote sustainable physical and psychological well-being among overweight and obese older adults, ultimately supporting more active, independent, and healthier aging.

## Materials and methods

### Study design

This randomized controlled trial was conducted at the Elderly Quality of Life Development Center, Nonthaburi City Municipality, Thailand, between March 2024 and October 2024. The study protocol was approved by the Ethical Review Committee for Research Involving Human Subjects, Health Science Group, Chulalongkorn University, Bangkok, Thailand (Approval No. 244/66). The trial was registered with the Chinese Clinical Trial Registry (ChiCTR; www.chictr.org.cn) on February 29, 2024 (Registration No. ChiCTR2400081386). All participants provided written informed consent prior to enrollment. Medical history and physical activity readiness were assessed using a standardized questionnaire. Randomization was performed by an independent researcher who was not involved in participant recruitment or outcome assessment, using a computerized random number generator. Group allocation was concealed using sealed, opaque envelopes. Due to the nature of the exercise intervention, blinding of participants, exercise instructors, and outcome assessors was not feasible. This limitation is acknowledged. All methods were performed in accordance with the Declaration of Helsinki and the Consolidated Standards of Reporting Trials (CONSORT) guidelines.

### Sample size calculation

The sample size was calculated using G*Power software (version 3.1.9.4). Body composition, specifically fat mass, was defined as the primary outcome and was therefore used as the basis for the sample size calculation. Based on an effect size (f = 0.40) for fat mass derived from a previous randomized controlled trial examining the effects of resistance exercise on body composition in obese older women [19], an a priori power analysis was conducted using an F-test for repeated-measures ANOVA with a within–between interaction. The parameters included an α error probability of 0.05, a power (1 − β) of 0.80, two groups,two repeated measurements, and an assumed correlation among repeated measures of 0.5. This analysis indicated that a minimum total sample size of 30 participants (15 per group) was required. To account for an anticipated dropout rate of approximately 27%, the target sample size was increased to 38 participants, resulting in 19 participants per group.

### Participants

**Fig 1:**
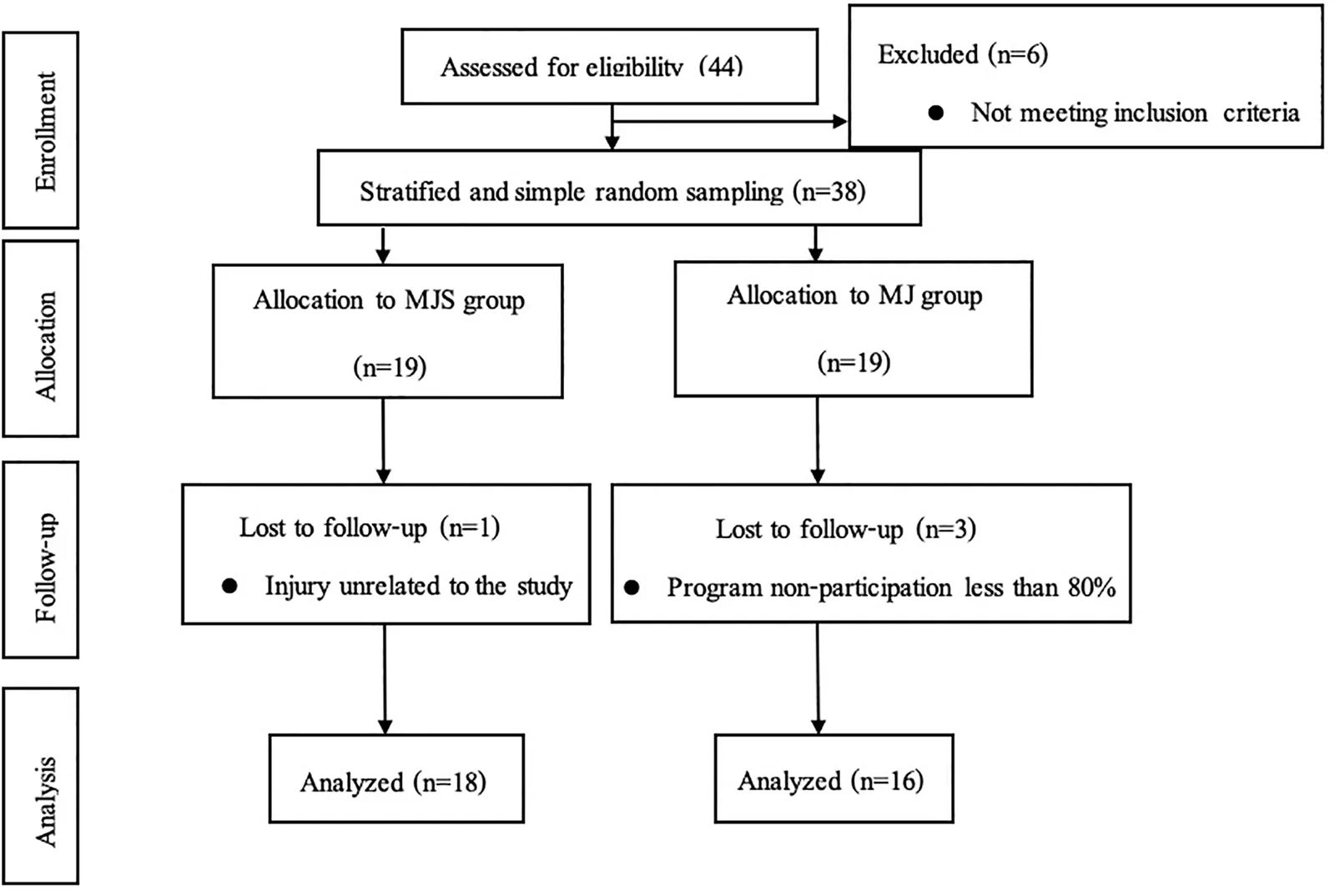
CONSORT flow diagram showing participant enrollment. A total of 44 older adults were screened for eligibility; 38 were randomized into the multi-joint self-efficacy (MJS) group (n = 19) and multi-joint (MJ) group (n = 19). After follow-up losses, 18 participants in the MJS group and 16 in the MJ group were included in the final analysis.

Participant flow is presented in Fig 1. A total of 38 overweight and obese older adults aged 60–75 years were initially recruited and randomly assigned to either the multi-joint exercise plus self-efficacy group (MJS; n = 19) or the multi-joint exercise group (MJ; n = 19). Following exclusions and dropouts during the intervention period, four participants withdrew from the study, resulting in an attrition rate of 11%. Consequently, 34 participants completed the study and were included in the final intention-to-treat analysis. In the final analytical sample, the MJS group comprised 18 participants (mean age 67.35 ± 3.69 years (mean ± SD); males/females = 2/16), while the MJ group comprised 16 participants (mean age 67.63 ± 5.28 years; males/females = 2/14).

All participants had a body mass index (BMI) ranging from 23 to 27.4 kg/m², classified according to Asian-specific BMI criteria [20]. Eligibility criteria included: (1) no history of uncontrolled diabetes, uncontrolled hypertension, cardiovascular disease, chronic obstructive pulmonary disease, or orthopedic conditions contraindicating exercise; (2) no use of weight-loss medication; (3) no participation in regular exercise or sports activities within the previous three months; and (4) low to moderate self-efficacy in performing multi-joint exercises, defined by questionnaire scores of 10–24 (low: 10–16; moderate: 17–24). Participants were excluded if they withdrew consent, experienced illness or injury that prevented continued participation, or attended fewer than 80% of the prescribed exercise sessions.

### Study protocol

Venous blood samples were collected before and after the intervention, following an 8-hour overnight fast. To control for diurnal variations in blood chemistry, all samples were taken at the same time of day for both the pre-test and post-test. Two hours after breakfast, participants completed the Thai-language versions of the Physical Activity Readiness Questionnaire (PAR-Q+) and a multi-joint exercise–specific self-efficacy questionnaire. Resting heart rate and blood pressure were measured while participants were seated, using a semi-automated monitor (Omron HEM-7120, Japan). Subsequently, assessments of body composition and physical function were conducted, in that order. During the intervention phase, participants in the MJ group performed only the multi-joint exercise program, while those in the MJS group participated in the same program with the addition of self-efficacy strategies.

### Intervention

The multi-joint exercise group (MJ group) participated in a 12-week program conducted three times per week, with each session lasting 60 min (10-min warm-up, 40-min exercise, and 10-min cool-down). All exercise sessions were supervised and delivered by the principal investigator together with trained research assistants holding master’s degrees in sports and exercise science, who were responsible for instructing participants, monitoring exercise technique, and ensuring adherence to the prescribed protocol. Each session included eight multi-joint exercises: (1) shoulder press with chair squats, (2) standing row with high knees, (3) triceps extension with chair squats, (4) standing front raise with hip extension, (5) calf raise with shrugs and chair squats, (6) front lunge with lateral arm raise, (7) standing leg curls with bicep curls, and (8) split squat with bicep curls. Exercises were performed for two sets of 10 repetitions, with progressive increases in training intensity over time.

Participants in the multi-joint exercise plus self-efficacy group (MJS group) received the same exercise program as the MJ group. In addition, self-efficacy–enhancing strategies based on Bandura’s self-efficacy theory were systematically integrated throughout the intervention. These strategies targeted the four primary sources of self-efficacy: (a) mastery experience, (b) vicarious experience, (c) verbal persuasion, and (d) emotional arousal, with the aim of enhancing participants’ confidence while performing the multi-joint exercises [7]. All outcome assessments were conducted jointly by the principal investigator and trained research assistants according to standardized assessment procedures. Intervention fidelity was monitored throughout the study. Adherence to the self-efficacy (SE) protocol was documented through structured observation and session attendance records, with ongoing communication via a dedicated LINE application group to support participant engagement and reinforce SE strategies. The self-efficacy strategies with multi-joint exercise protocol showed in Table 1.

**Table 1:** A 12-week program combining progressive multi-joint exercises with self-efficacy strategies to enhance exercise adherence and confidence.

Week(s)	Multi-joint exercise	Self-efficacy strategies
1–2	8 repetitions, 2 sets, 1-minute rest between sets and exercises. Equipment: Women (1 kg dumbbell, 0.5 kg sandbag); Men (1.5 kg dumbbell, 1 kg sandbag)	Mastery experience: Exercise education and discussion of past exercise experiences to promote confidence.
3–4	10 repetitions, same rest interval and sets.	Mastery experience (continued): Reinforcing skills and promoting belief in exercise capability.
5–6	Increased resistance: Women (1.5 kg dumbbell, 1 kg sandbag); Men (2 kg dumbbell, 1.5 kg sandbag)	Mastery experience and guided sessions: Instructor-led activities with researcher guidance and feedback.
7–8	Increased to 3 sets, same resistance.	Mastery and vicarious experience: Introduction to role modeling by peers.
9–12	Increased resistance: Women (1.5 kg dumbbell, 1.5 kg sandbag); Men (2 kg dumbbell, 2 kg sandbag)	Vicarious experience and self-modeling: Peer leadership and participant role models to reinforce efficacy.
1–12	Sessions were supervised by an external instructor with researcher support, while participants engaged in self-monitoring through exercise diaries. Verbal persuasion and emotional arousal were facilitated via encouragement, motivational messages, and observation of affective responses.	
5–12	Behavioral reinforcement was provided through structured praise and social rewards recognizing leadership and effort.	
6–12	Peer-led sessions facilitated vicarious experience through observation and interaction with successful role models.	

### Measurement

Outcome measures were designed to evaluate self-efficacy related to multi-joint exercise, with body composition defined as the primary outcome, and physical function and blood biochemistry evaluated as secondary outcomes. All assessments were conducted at baseline (week 0) and post-intervention (week 12). All assessments of self-efficacy, body composition, and physical function were performed by trained research assistants under the supervision of the principal investigator, following standardized and validated protocols. Blood chemistry measurements were obtained by two certified medical technologists from the Faculty of Allied Health Sciences, Chulalongkorn University, who were responsible for venipuncture and blood sample collection.

#### Multi-joint exercise capability self-efficacy questionnaire.

The reliability of a self-efficacy capability questionnaire for designing multi-joint exercise programs in overweight and obese older adults was evaluated. Tested in a sample of 40 participants, the questionnaire demonstrated high internal consistency (Cronbach’s α = 0.90). Subscale reliability coefficients were 0.72 for knowledge, 0.88 for self-efficacy capability, and 0.76 for outcome expectation, confirming the questionnaire’s reliability for assessing exercise planning capability. The assessment criteria were based on an adapted criterion-referenced approach following Bloom’s principles and evaluation methods [21]. Detailed scoring procedures and level definitions are provided in the Supplementary Material.

#### Body composition.

Body composition was evaluated using a bioelectrical impedance analyzer (BIA), specifically the Omron HBF-375, Japan measuring parameters such as percentage body fat, fat mass, fat-free mass, and muscle mass [22].

#### Physical function.

Physical function was assessed using a standardized battery of tests, including measures of muscle strength, muscular endurance, balance, and mobility.

#### Muscle strength.

Muscle strength was assessed for both the upper and lower body using a handgrip dynamometer and a back-and-leg dynamometer (Takei Hand Grip Dynamometer T.K.K.5401 and Takei Back and Leg Dynamometer T.K.K.5002, respectively; Takei Scientific Instruments, Japan). Each test was performed twice, and the best value was recorded [23].

#### Muscular endurance.

Upper-body muscular endurance was assessed using the 30-second biceps curl test with dumbbells. According to the standard protocol, women use a 5-lb (2.2 kg) dumbbell and men use an 8-lb (3.63 kg) dumbbell. In the present study, female participants used a 2-kg dumbbell and male participants used a 4-kg dumbbell. The total number of correctly completed repetitions was recorded [24].

Lower-body muscular endurance was evaluated using the 30-second chair stand test, in which participants were instructed to repeatedly rise from and return to a seated position on a chair for 30 seconds. The total number of correctly completed stands was recorded as an indicator of lower-body endurance [24].

#### Agility and dynamic balance testing.

Agility and dynamic balance were assessed using the 3-m Up-and-Go test. Participants were instructed to stand up from a seated position, walk a distance of 3 meters, turn around, walk back to the chair, and sit down as quickly and safely as possible. Performance was recorded as the time required to complete the task, measured in seconds [25].

#### Blood Collection and Biochemical Analysis.

Venous blood samples were collected by a certified medical technologist from the antecubital vein after an overnight fasting period of at least 8 hours. Blood for glucose and lipid profile analyses was collected into serum separator tubes, while blood for HbA1c analysis was collected into dipotassium ethylenediaminetetraacetic acid (K_2_EDTA) tubes. All biochemical analyses were performed immediately following collection at a certified clinical laboratory within the Faculty of Allied Health Sciences, Chulalongkorn University, Pathumwan, Thailand.

Fasting plasma glucose (FPG) and lipid profiles (total cholesterol, triglycerides, high-density lipoprotein cholesterol (HDL-c), and low-density lipoprotein cholesterol (LDL-c)) were analyzed using an automated chemistry analyzer (Beckman Coulter AU480, USA) employing enzymatic colorimetric methods. The intra-assay coefficient of variation (CV) for glucose and lipid measurements was less than 3%. Glycated hemoglobin (HbA1c) was measured using high-performance liquid chromatography (HPLC) on a Bio-Rad D-10™ Hemoglobin Testing System (Bio-Rad Laboratories, USA). The intra-assay CV for HbA1c measurement was below 2%. High-sensitivity C-reactive protein (hs-CRP) was assessed via an immunoturbidimetric assay using the same chemistry analyzer (Beckman Coulter AU480). The intra-assay CV for hs-CRP was less than 5%. All assays were performed according to the manufacturers’ protocols and quality control standards. Blood samples were processed and analyzed within two hours of collection to ensure data accuracy.

## Statistical analysis

Statistical analyses were performed using SPSS version 26 (IBM Corp., Armonk, NY, USA). All randomized participants were included in the analyses according to the intention-to-treat principle. Changes over time and between-group differences were analyzed using linear mixed-effects models fitted by restricted maximum likelihood (REML), with fixed effects for group, time, and the group × time interaction. Participants were included as a random intercept to account for within-subject correlation across repeated measurements, and a first-order autoregressive [AR(1)] covariance structure was specified for repeated observations. Linear mixed-effects models were chosen because they allow the inclusion of all available data and appropriately handle missing post-test data resulting from participant dropout under the assumption of missing at random, without the need for data imputation. Estimated marginal means (EMMs), standard errors (SEs), and 95% confidence intervals (CIs) were obtained from the fitted models and are presented for each group at each time point. When significant effects were identified, Bonferroni-adjusted pairwise comparisons of the estimated marginal means were performed to compare pre- and post-intervention values within each group. The statistical significance of intervention effects was evaluated using the group × time interaction term. Statistical significance was determined using two-tailed tests with p < 0.05.

## Results

### Participant flow and analysis approach

All randomized participants were included in the analyses under the intention-to-treat principle. Outcomes were analyzed using linear mixed-effects models with fixed effects for group, time, and group × time interaction, and random intercepts for participants. Estimated marginal means (EMMs) with standard errors (SEs) and 95% confidence intervals (CIs) are reported.

### Physiological characteristics and body composition

Estimated marginal means for physiological characteristics and body composition outcomes are presented in Table 2.

**Table 2:** Estimated marginal means of physiological characteristics and body composition by group and time derived from linear mixed-effects models.

Variables	Group	Pre (EMM ± SE)	95%CI	Post (EMM ± SE)	95%CI	Change EMM	p-value Time	p-value Group	p-value Time × Group
HR at rest (beats·min^-1^)	MJS	73.5 ± 2.1	69.2-77.8	74.3 ± 2.2	69.8-78.7	0.8	0.002	0.483	0.009
	MJ	67.9 ± 2.3	63.4-72.4	75.7 ± 2.3*	71.0-80.4	7.8			
SBP at rest (mmHg)	MJS	128.8 ± 3.2	122.4-135.2	124.9 ± 3.4	118.2-131.6	−3.9	0.925	0.619	0.091
	MJ	126.7 ± 3.4	119.9-133.5	131.1 ± 3.5	134.0-138.2	4.4			
DBP at rest (mmHg)	MJS	78.6 ± 2.2	74.2-82.9	76.7 ± 2.2	72.2-81.2	−1.9	0.987	0.895	0.150
	MJ	77.1 ± 2.3	72.5-81.7	78.9 ± 2.4	74.2-83.7	1.8			
Body mass (kg)	MJS	61.3 **±** 1.8	57.7-64.9	59.7 ± 1.8*	56.1-63.4	−1.6	<0.001	0.315	0.412
	MJ	63.7 **±** 1.9	59.7-67.5	62.6 ± 1.9	58.8-66.4	−1.1			
BMI (kg·m^-2^)	MJS	25.3 ± 0.6	24.1-26.5	24.6 ± 0.6*	23.4-25.8	−0.7	<0.001	0.600	0.095
	MJ	25.5 ± 0.6	24.3-26.8	25.3 ± 0.6	24.0-26.5	−0.2			
Fat mass (kg)	MJS	21.9 ± 1.0	20.0-23.9	20.7 ± 1.0*	18.7-22.7	−1.2	<0.001	0.249	0.274
	MJ	23.3 ± 1.0	21.3-25.4	22.6 ± 1.0	20.5-25.7	−0.7			
Body fat (%)	MJS	35.7 ± 0.8	34.2-37.2	34.5 ± 0.8*	32.9-36.0	−1.2	0.007	0.320	0.427
	MJ	36.5 ± 0.8	34.9-38.1	35.8 ± 0.8	34.2-37.4	−0.7			
Fat-free mass (kg)	MJS	39.4 ± 1.0	37.3-41.5	39.0 ± 1.0	36.9-41.1	−0.4	0.224	0.512	0.924
	MJ	40.3 ± 1.1	38.1-42.6	40.0 ± 1.1	37.7-42.2	−0.3			
Muscle mass (kg)	MJS	14.0 ± 0.5	13.0-15.0	13.9 ± 0.5	12.9-14.9	−0.1	0.298	0.627	0.886
	MJ	14.4 ± 0.5	13.3-15.4	14.2 ± 0.5	13.1-15.2	−0.2			

Values are estimated marginal means (EMM) ± standard error (SE) with 95% confidence intervals (CIs), derived from linear mixed-effects models. Pairwise comparisons between pre- and post-intervention within each group were adjusted using the Bonferroni correction. *P < 0.05 indicates a statistically significant pre- to post-intervention change within the same group after Bonferroni adjustment. P-values for Time, Group, and Group × Time represent the fixed effects estimated from the linear mixed-effects model. Abbreviations: MJS, multi-joint exercise combined with self-efficacy theory; MJ, multi-joint exercise; HR, heart rate; SBP, systolic blood pressure; DBP, diastolic blood pressure; BMI, body mass index.

### Physiological characteristics and body composition

Table 2 presents the estimated marginal means (EMMs) of physiological characteristics and body composition before and after the 12-week intervention.

**Fig 2:**
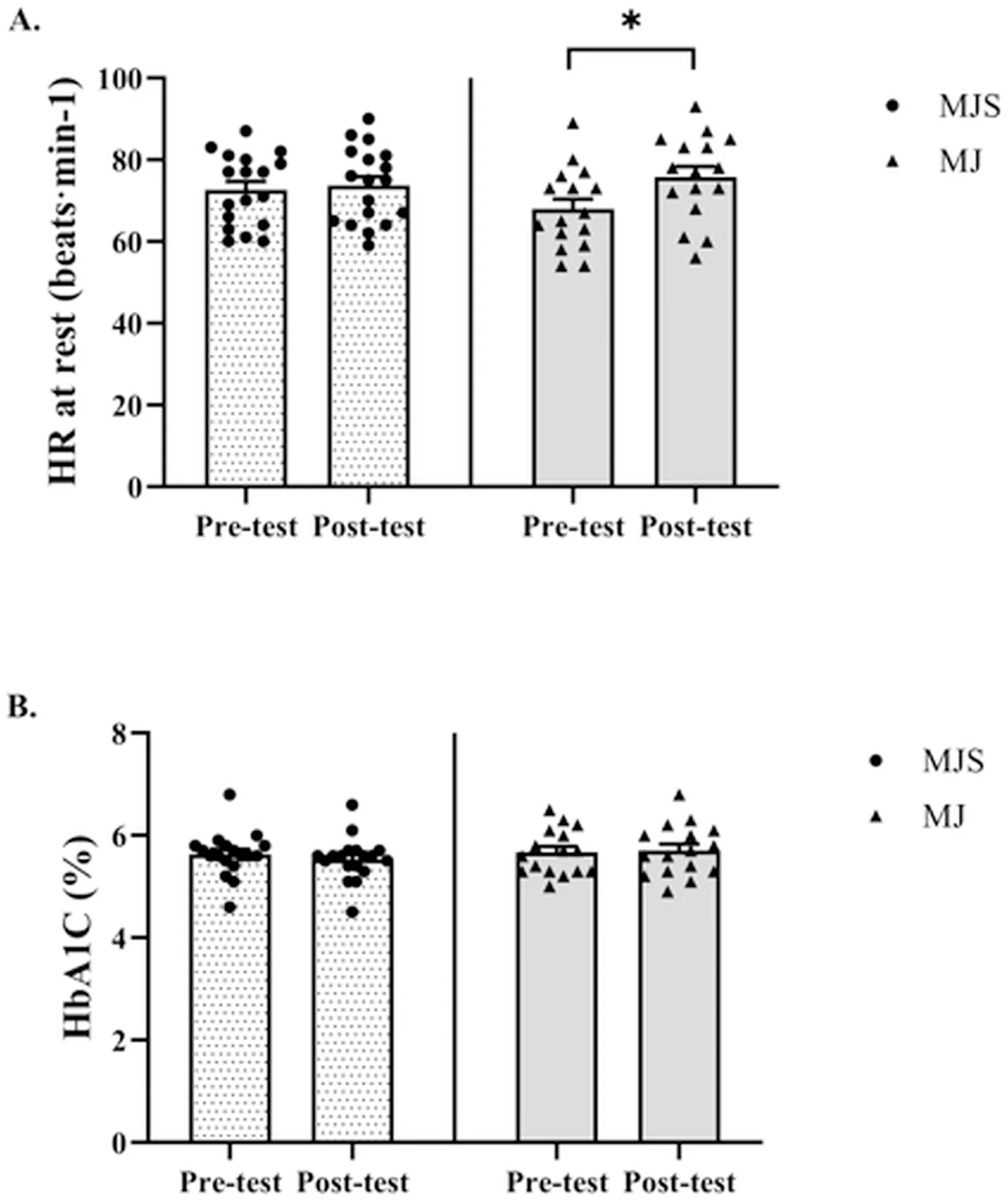
Changes in resting heart rate (HR) and glycated hemoglobin (HbA1c) following the 12-week intervention. (A) Resting heart rate (HR). (B) Glycated hemoglobin (HbA1c). Values are presented as estimated marginal means (EMMs) ± standard errors (SEs), with individual participant data overlaid. MJS = multi-joint exercise combined with a self-efficacy intervention; MJ = multi-joint exercise only. Pre-test = baseline assessment before the intervention; Post-test = assessment after the 12-week intervention. *P < 0.05 indicates a statistically significant pre- to post-intervention change within the same group.

A significant group × time interaction was observed only for resting heart rate (HR) (p = 0.009) (Fig 2A). Bonferroni-adjusted pairwise comparisons demonstrated a significant increase in resting HR in the MJ group from pre- to post-intervention (67.9 ± 2.3 to 75.7 ± 2.3 beats·min ⁻ ¹,), whereas no significant change was observed in the MJS group.

No significant group × time interactions were found for systolic blood pressure (SBP; p = 0.091), diastolic blood pressure (DBP; p = 0.150), body mass (p = 0.412), body mass index (BMI; p = 0.095), fat mass (p = 0.274), body fat percentage (p = 0.427), fat-free mass (p = 0.924), or muscle mass (p = 0.886). However, Bonferroni-adjusted pairwise comparisons revealed significant within-group reductions in body mass, BMI, fat mass, and body fat percentage in the MJS group. No significant within-group changes were observed in the MJ group for these variables. Fat-free mass and muscle mass did not change significantly in either group following the intervention.

### Physical function outcomes

Table 3 presents the estimated marginal means (EMMs) of physical function outcomes before and after the 12-week intervention. No significant group × time interactions were observed for right-hand grip strength (p = 0.642), left-hand grip strength (p = 0.458), back strength (p = 0.582), leg strength (p = 0.690), right-arm 30-s arm curl (p = 0.223), left-arm 30-s arm curl (p = 0.583), 30-s chair stand test (p = 0.299), single-leg stance (p = 0.718), or Timed Up and Go test (p = 0.744).

**Table 3:** Estimated marginal means of physical function outcomes by group and time derived from linear mixed-effects models.

Variables	Group	Pre (EMM ± SE)	95%CI	Post (EMM ± SE)	95%CI	Change EMM	p-value Time	p-value Group	p-value Time × Group
Hand grip									
Right hand (kg)	MJS	24.3 ± 1.1	22.1-26.4	25.6 ± 1.1*	23.4-27.7	1.3	0.004	0.935	0.642
	MJ	23.9 ± 1.1	21.6-26.1	25.7 ± 1.1*	23.4-28.0	1.8			
Left hand (kg)	MJS	22.2 ± 1.1	19.9-24.4	24.0 ± 1.1*	21.7-26.3	1.8	0.033	0.903	0.458
	MJ	22.8 ± 1.2	20.5-25.1	23.7 ± 1.2*	21.3-26.1	0.9			
Back and Leg dynamometer									
Back strength (kg)	MJS	44.8 ± 2.8	39.3-50.3	54.5 ± 2.9*	48.8-60.3	9.7	<0.001	0.808	0.582
	MJ	44.4 ± 2.9	38.6-50.3	56.6 ± 3.0*	50.5-62.7	12.2			
Leg strength (kg)	MJS	60.9 ± 6.6	47.5-74.2	64.2 ± 6.8	50.5-77.9	3.3	0.233	0.388	0.690
	MJ	51.4 ± 7.0	37.4-65.5	58.1 ± 7.2	43.6-72.6	6.7			
30-sec arm curl test									
Right arm (rep)	MJS	20.6 ± 1.4	17.8-23.4	32.5 ± 1.4*	29.6-35.4	11.9	<0.001	0.272	0.223
	MJ	20.0 ± 1.5	17.1-22.9	29.2 ± 1.5*	26.1-32.2	9.2			
Left arm (rep)	MJS	22.0 ± 1.5	19.0-25.0	33.9 ± 1.6*	30.8-37.0	11.9	<0.001	0.120	0.583
	MJ	19.8 ± 1.6	16.7-23.0	30.3 ± 1.6*	27.0-33.6	10.5			
30-sec chair stand test (rep)	MJS	42.3 ± 2.1	38.1-46.5	49.9 ± 2.2*	45.5-54.3	7.6	<0.001	0.098	0.299
	MJ	39.2 ± 2.2	34.8-43.7	43.7 ± 2.3*	39.0-48.3	4.5			
Single-leg stance (sec)	MJS	41.0 ± 6.8	27.4-54.7	51.5 ± 7.1*	37.3-65.6	10.5	0.019	0.188	0.718
	MJ	27.4 ± 7.2	13.1-41.8	41.5 ± 7.5*	26.5-56.5	14.1			
Timed Up and Go test (sec)	MJS	7.9 ± 0.2	7.5-8.3	6.5 ± 0.2*	6.0-6.9	-1.4	<0.001	0.536	0.744
	MJ	8.0 ± 0.2	7.5-8.5	6.7 ± 0.2*	6.2-7.2	-1.3			

Values are presented as estimated marginal means (EMMs) ± standard errors (SEs) with 95% confidence intervals (CIs), derived from linear mixed-effects models fitted using restricted maximum likelihood (REML) under the intention-to-treat principle. Change EMM represents the estimated within-group change from pre- to post-intervention based on the fitted linear mixed-effects model. *P < 0.05 indicates a statistically significant pre- to post-intervention change within the same group after Bonferroni adjustment. P-values for Time, Group, and Group × Time represent the fixed effects estimated from the linear mixed-effects model. P < 0.05 was considered statistically significant. Abbreviations: MJS, multi-joint exercise combined with self-efficacy theory; MJ, multi-joint exercise

Despite the absence of significant group × time interactions, Bonferroni-adjusted pairwise comparisons revealed significant within-group improvements in both groups for right-hand grip strength, left-hand grip strength, back strength, right-arm 30-s arm curl, left-arm 30-s arm curl, 30-s chair stand, single-leg stance, and Timed Up and Go. No significant within-group changes were observed for leg strength in either group following the intervention.

### Blood chemistry outcomes

Table 4 presents the estimated marginal means (EMMs) of blood chemistry outcomes before and after the 12-week intervention.

**Table 4:** Estimated marginal means of blood chemistry outcomes by group and time derived from linear mixed-effects models.

Variables	Group	Pre (EMM ± SE)	95%CI	Post (EMM ± SE)	95%CI	Change EMM	p-value Time	p-value Group	p-value Time × Group
Total cholesterol (mg/dL)	MJS	231.5 ± 10.4	210.6-252.4	209.9 ± 11.0	188.3-231.4	−21.6	0.214	0.475	0.056
	MJ	208.3 ± 11.0	186.2-230.4	213.0 ± 11.4	190.2-235.9	4.7			
Triglyceride (mg/dL)	MJS	115.0 ± 9.3	96.4-133.6	127.5 ± 9.5	108.4-146.7	12.5	0.004	0.775	0.352
	MJ	106.0 ± 9.8	86.4-125.6	129.4 ± 10.1*	109.1-149.6	23.4			
HDL-c (mg/dL)	MJS	57.6 ± 2.8	52.0-63.1	54.3 ± 2.9	48.6-59.9	−3.3	0.389	0.304	0.201
	MJ	59.5 ± 2.9	53.7-65.3	60.2 ± 3.0	54.2-66.1	0.7			
LDL-c (mg/dL)	MJS	151.0 ± 9.4	132.1-169.9	130.0 ± 9.7	110.5-149.5	−21.0	0.076	0.298	0.092
	MJ	127.5 ± 9.9	107.6-147.4	126.9 ± 10.3	103.3-147.5	−0.6			
FPG (mg/dL)	MJS	92.2 ± 3.0	86.1-98.2	93.7 ± 3.1	87.6-99.9	1.5	0.007	0.103	0.104
	MJ	97.0 ± 3.2	90.6-103.4	103.0 ± 3.2*	96.4-109.5	6.0			
HbA1c (%)	MJS	5.7 ± 0.1	5.5-5.9	5.6 ± 0.1	5.4-5.8	−0.1	0.270	0.881	0.014
	MJ	5.6 ± 0.1	5.4-5.9	5.7 ± 0.1	5.5-5.9	0.1			
hs-CRP (mg/L)	MJS	1.8 ± 0.5	0.9-2.7	1.1 ± 0.5	0.1-2.0	−0.7	0.985	0.163	0.123
	MJ	1.7 ± 0.5	0.8-2.7	2.5 ± 0.5	1.5-3.5	0.8			

Values are presented as estimated marginal means (EMMs) ± standard errors (SEs) with 95% confidence intervals (CIs), derived from linear mixed-effects models fitted using restricted maximum likelihood (REML) under the intention-to-treat principle. Change EMM represents the estimated within-group change from pre- to post-intervention based on the fitted linear mixed-effects model. Pairwise comparisons between pre- and post-intervention within each group were adjusted using the Bonferroni correction. P-values for Time, Group, and Group × Time represent the fixed effects estimated from the linear mixed-effects model. *P < 0.05 indicates a statistically significant pre- to post-intervention change within the same group after Bonferroni adjustment. Abbreviations: MJS, multi-joint exercise combined with self-efficacy theory; MJ, multi-joint exercise; HDL-c, high-density lipoprotein cholesterol; LDL-c, low-density lipoprotein cholesterol; FPG, fasting plasma glucose; HbA1c, glycated hemoglobin; hs-CRP, high-sensitivity C-reactive protein.

A significant group × time interaction was observed for HbA1c (p = 0.014) (Fig 2B). However, Bonferroni-adjusted pairwise comparisons did not demonstrate statistically significant pre- to post-intervention changes within either group. No significant group × time interactions were observed for total cholesterol (p = 0.056), triglycerides (p = 0.352), HDL-c (p = 0.201), LDL-c (p = 0.092), fasting plasma glucose (FPG; p = 0.104), or hs-CRP (p = 0.123). In contrast, Bonferroni-adjusted pairwise comparisons demonstrated a significant increase in triglyceride levels and fasting plasma glucose in the MJ group. No significant within-group changes were observed for total cholesterol, HDL-c, LDL-c, or hs-CRP in either group.

### Psychological outcomes (Knowledge, Self-efficacy, Expectation)

**Fig 3:**
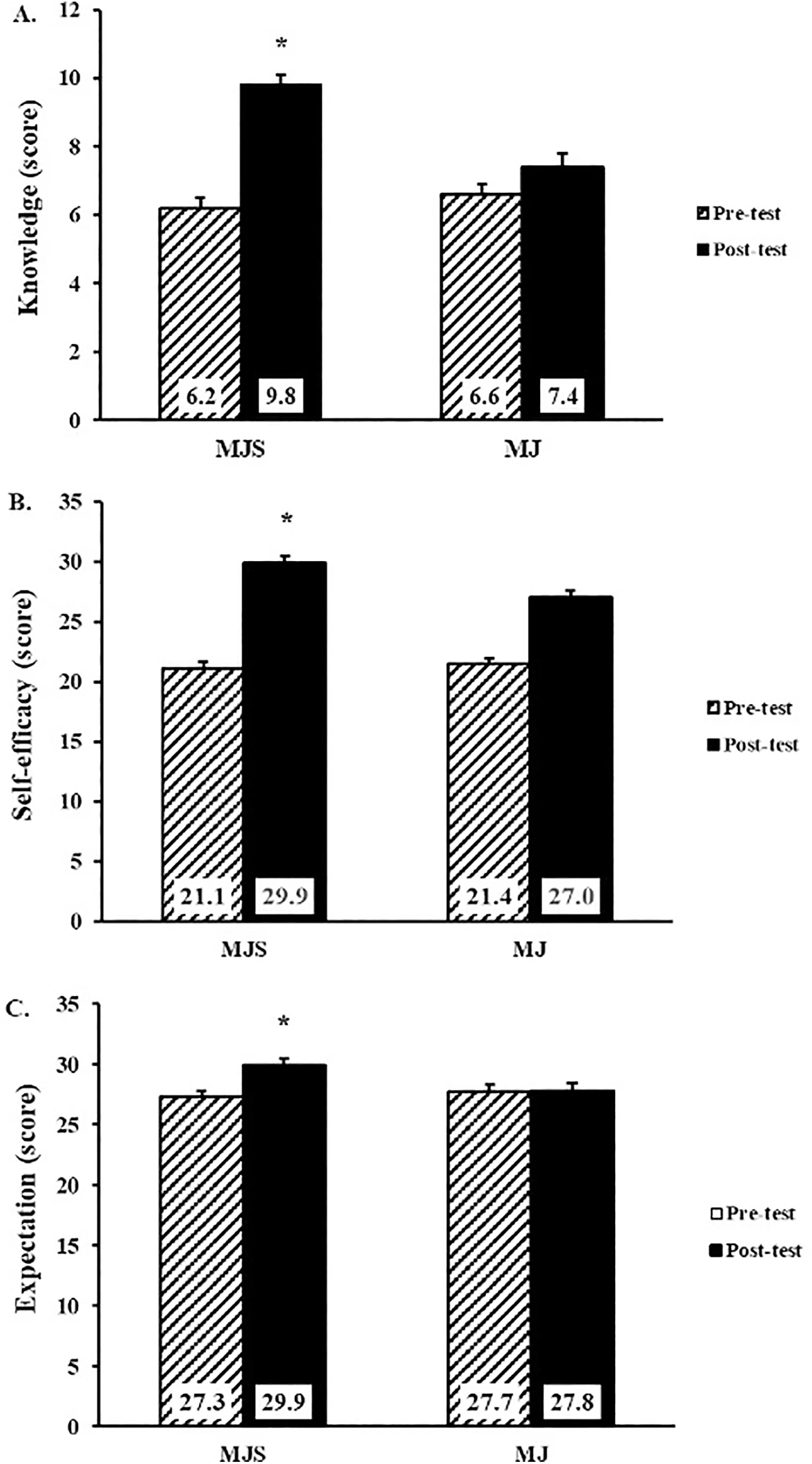
Estimated marginal means of knowledge (A), self-efficacy (B), and outcome expectation (C) scores at pre- and post-intervention in the MJS and MJ groups. Values are estimated marginal means ± standard error. * p < 0.05 indicates a significant time × group interaction derived from linear mixed-effects models.

Estimated marginal means for psychological outcomes are presented in (Fig 3) Significant group × time interactions were observed for knowledge, self-efficacy, and outcome expectation, indicating differential changes between groups over time. For knowledge, a significant group × time interaction was observed (p < **0.001).** The MJS group demonstrated a marked increase from pre- to post-intervention **(6.2 ± 0.3** to **9.8 ± 0.3),** whereas the MJ group showed a smaller increase **(6.6 ± 0.3** to **7.4 ± 0.4), indicating a greater improvement in knowledge in the MJS group. Similarly, self-efficacy showed a significant group × time interaction (p = 0.003).** Participants in the MJS group exhibited a larger increase in self-efficacy scores **(21.1 ± 0.5** to **29.9 ± 0.5)** compared with the MJ group **(21.4 ± 0.5** to **27.0 ± 0.6).** For outcome expectation, a significant group × time interaction was also observed (p = **0.020).** The MJS group demonstrated an increase from **27.3 ± 0.5** to **29.9 ± 0.6,** whereas the MJ group showed minimal change over time (**27.7 ± 0.6** to **27.8** ± **0.6).**

Overall, these findings indicate that integrating self-efficacy strategies into multi-joint exercise was associated with greater improvements in psychological outcomes compared with multi-joint exercise alone.

## Discussion

The present study provides supportive evidence that integrating self-efficacy strategies into a multi-joint exercise program may be associated with more favorable psychological and selected physiological outcomes in overweight and obese older adults compared with multi-joint exercise alone. Both interventions were associated with improvements in physical function, whereas the addition of self-efficacy strategies was particularly associated with greater improvements in knowledge, self-efficacy, and outcome expectations.

With respect to physiological characteristics, a significant group × time interaction was observed for resting heart rate, with minimal change in the MJS group and an increase in the MJ group. This pattern may suggest an association between the integration of self-efficacy strategies and the maintenance of resting heart rate during the intervention; however, the physiological mechanisms underlying this finding were not directly assessed and remain uncertain. Emotional self-efficacy has previously been associated with autonomic regulation and may help buffer autonomic nervous system dysregulation [26]. Nevertheless, the present findings do not establish a causal mechanism, and the non-significant group × time interaction observed for systolic blood pressure should be interpreted cautiously.

For body composition, significant within-group reductions in body mass, body mass index, fat mass, and body fat percentage were observed in the MJS group; however, no significant group × time interactions were identified for these outcomes. Thus, although favorable within-group changes occurred in the MJS group, the findings do not demonstrate that the addition of self-efficacy strategies produced greater body composition changes than multi-joint exercise alone. Longer intervention durations, higher training volumes, or dietary modification may be required to produce clearer between-group differences in body composition among older adults [27].

Regarding physical function, both groups demonstrated improvements across a range of outcomes, including muscle strength, muscular endurance, functional lower-limb performance, balance, and mobility. However, no significant time × group interactions were observed for any physical function measures. These results suggest that multi-joint exercise itself is effective in improving functional capacity in older adults, regardless of whether self-efficacy strategies are included [28,29]. Such improvements are plausibly attributable to the neuromuscular and functional demands of resistance-based multi-joint exercise, which can enhance strength, coordination, and functional independence [30,31].

In contrast, more pronounced group differences were observed for metabolic outcomes. A significant group × time interaction was observed for HbA1c. However, Bonferroni-adjusted pairwise comparisons did not identify statistically significant pre- to post-intervention changes within either group. Accordingly, the HbA1c finding should be interpreted as evidence of differing patterns of change between groups rather than as confirmation of a significant reduction or increase within either group. No significant group × time interactions were observed for fasting plasma glucose or hs-CRP. Because the metabolic outcomes were exploratory, the sample size was modest, and dietary intake, medication adherence, and habitual physical activity outside the supervised sessions were not strictly controlled, these findings should be interpreted cautiously. The most consistent between-group differences were observed for the psychological outcomes. Significant group × time interactions were identified for knowledge, self-efficacy, and outcome expectations, with the MJS group demonstrating greater improvements than the MJ group. These findings are consistent with self-efficacy theory, which emphasizes the roles of self-efficacy and outcome expectations in shaping health behaviors [7]. Greater knowledge and confidence may strengthen individuals’ beliefs in their ability to initiate and maintain exercise participation, thereby reinforcing positive behavioral patterns [32,33]. Although the present study did not directly test the behavioral mechanisms linking psychological and physiological responses, the findings support the potential value of incorporating theory-based self-efficacy strategies into exercise programs for older adults.

Collectively, the findings suggest that integrating self-efficacy strategies into multi-joint resistance exercise may provide complementary psychological benefits and may be associated with selected physiological outcomes. However, these observations require confirmation in larger, adequately powered randomized controlled trials. These findings have important implications for the design of exercise interventions targeting older populations, highlighting the potential value of incorporating theory-based psychological components to enhance the overall effectiveness of exercise programs.

Limitations should be acknowledged. Participants were recruited voluntarily from a community setting, and the study used a two-group design without either a non-exercise control group or an attention-matched control group. Consequently, the observed psychological improvements may partly reflect non-specific effects of researcher attention or social interaction. The predominance of female participants may limit the generalizability of the findings to older males. In addition, because eligibility was restricted to older adults with low-to-moderate baseline self-efficacy, the findings may not generalize to individuals with high baseline self-efficacy. Body composition was assessed using bioelectrical impedance analysis (BIA), which is practical for community-based research but is less precise than dual-energy X-ray absorptiometry (DXA).

## Conclusion

This study showed that multi-joint resistance exercise improves physical function in overweight and obese older adults, irrespective of the inclusion of self-efficacy strategies. Although body composition (fat mass) was the primary outcome, the addition of self-efficacy strategies did not result in significant between-group differences in body composition. Integrating theory-based self-efficacy strategies was associated with greater improvements in psychological outcomes and selected physiological outcomes. Findings related to metabolic outcomes, particularly HbA1c, should be interpreted cautiously. Future studies should confirm these findings in larger, adequately powered, and longer-term trials and examine implementation outcomes.

## Supporting information

S1 File Study Questionnaire. Questionnaire used in the study.
(PDF)

S2 File CONSORT 2010 Checklist. Completed CONSORT 2010 checklist for the randomized controlled trial.
(PDF)

S3 File Trial Protocol. Trial protocol for this randomized controlled trial.
(PDF)
