# Supplementary material for: Effects of integrating self-efficacy strategies into multi-joint resistance exercise for overweight and obese older adults: A randomized controlled trial
Source: PLoS One. 2026 Aug 21;21(8):e0335830. doi: 10.1371/journal.pone.0335830 (PMC13496450; doi:10.1371/journal.pone.0335830)
Supplement: Supplementary file 1 [file pone.0335830.s001.pdf]

The questionnaire consists of 4 parts were as follows:

The first part gathered general and health information, including name, age, underlying diseases, and self-care through exercise.

The second part assessed knowledge about multi-joint exercises using a multiple-choice format, with a total of 10 questions. Each question had two answer choices: "Yes" and "No." The scoring criteria are as follows: answering "Yes" earns 1 point, while answering "No" earns 0 points. A low level is 0–4.9, a medium level is 5–7.9, and a high level is 8–10.

The third part assessed self-efficacy in multi-joint exercises, and the fourth part assessed perceived outcomes of multiple-choice exercises, both using a multiple-choice format with a total of 10 questions. Each question had three answer choices: "Yes," "Unsure," and "No." The questions included both positive and negative statements. The scoring criteria are as follows: for positive statements, answering "Yes" earns 3 points, "Unsure" earns 2 points, and "No" earns 1 point; for negative statements, the scoring is reversed: "Yes" earns 1 point, "Unsure" earns 2 points, and "No" earns 3 points. The scoring range is 10–30 points, categorized into three levels: a low level (10–16), a medium level (17–24), and a high level (25–30).
