# Supplementary material for: Effects of integrating self-efficacy strategies into multi-joint resistance exercise for overweight and obese older adults: A randomized controlled trial
Source: PLoS One. 2026 Aug 21;21(8):e0335830. doi: 10.1371/journal.pone.0335830 (PMC13496450; doi:10.1371/journal.pone.0335830)
Supplement: Supplementary file 3 [file pone.0335830.s003.pdf]

Part 1: Research Protocol

1. Project Summary

Overweight and obesity among older adults have become major public health concerns worldwide and are associated with increased risks of cardiometabolic diseases, functional decline, frailty, and reduced quality of life. Progressive resistance exercise is an effective intervention for improving muscle strength, body composition, and physical function in older adults. However, long-term adherence to exercise programs remains challenging because psychological factors, particularly low exercise self-efficacy, frequently limit sustained participation.

Self-efficacy, derived from Social Cognitive Theory, is one of the strongest predictors of exercise initiation and maintenance. Strategies designed to enhance self-efficacy, including mastery experiences, verbal persuasion, vicarious experience, and emotional regulation, may improve adherence and promote greater physiological adaptations when integrated into structured exercise programs.

Although resistance training and self-efficacy interventions have individually demonstrated beneficial effects, evidence regarding their combined effects in overweight and obese older adults remains limited. Therefore, this randomized controlled trial investigates whether integrating self-efficacy strategies into a progressive multi-joint resistance exercise program provides additional benefits beyond exercise alone.

2. General Information

2.1 Protocol Title

Effects of Integrating Self-Efficacy Strategies into Multi-Joint Resistance Exercise for Overweight and Obese Older Adults: A Randomized Controlled

2.2 Protocol ID ChiCTR2400081386

2.2 Date of Registration February 29, 2024

2.3 Sponsor

This study is supported by the 90th Anniversary of Chulalongkorn University, Rachadapisek Sompote Fund and the Faculty of Sports Science Fund, Chulalongkorn University.

Principal Investigator; responsibilities of each

Oratai Kanlayawut[1]; data collection, analysis and interpretation of results, writing (original draft), writing (review and editing)., Raweewan Maphong[1]; writing (review and editing)., Waris Wongpipit[2,3,4]; writing (review and editing)., Thanomwong Kritpet[1*]; conceptualization, study design, supervision, writing (original draft), writing (review and editing).. [1]Faculty of Sports Science, Chulalongkorn University, Bangkok, Thailand; [2]Division of Health and Physical Education, Faculty of Education, Chulalongkorn University, Bangkok, Thailand; [3]Department of Sports Science and Physical Education, Faculty of Education, The Chinese University of Hong Kong, Hong Kong S.A.R.; [4]Centre of Excellence in Educational Invention and Innovation, Faculty of Education, Chulalongkorn University, Bangkok, Thailand

2.4 Site Senior Citizens' Quality of Life Development Center, Nonthaburi City Municipality

2.5 Laboratory Faculty of Allied Health Sciences, Chulalongkorn University

3. Rationale and Background Information

The prevalence of overweight and obesity among older adults has increased substantially during recent decades. Excess adiposity contributes to impaired mobility, reduced muscle function, insulin resistance, hypertension, dyslipidemia, chronic low-grade inflammation, and increased cardiovascular risk. These changes accelerate functional decline and reduce independence during aging.

Resistance exercise is widely recommended for older adults because it improves muscle strength, preserves lean body mass, reduces fat mass, and enhances physical performance. Multi-joint resistance exercise simultaneously activates multiple muscle groups during functional movement patterns and has demonstrated favorable effects on strength, mobility, and overall physical function.

Despite these physiological benefits, maintaining long-term participation remains difficult. Exercise adherence is strongly influenced by psychological factors, particularly self-efficacy. Individuals with greater confidence in their ability to exercise are more likely to initiate, maintain, and progressively increase physical activity levels.

Bandura's Social Cognitive Theory identifies four principal sources of self-efficacy: Mastery experience, vicarious experience, verbal persuasion, regulation of physiological and emotional states

Integrating these evidence-based strategies into supervised resistance exercise may enhance motivation, improve adherence, and produce greater physiological and psychological benefits than exercise alone.

Although previous studies have investigated resistance training and behavioral interventions separately, few randomized controlled trials have evaluated their combined effects in overweight and obese older adults. This study therefore aims to address this gap.

4. References

(References from the manuscript would be listed here as per journal requirements.)

5. Study Goals and Objectives

5.1 Primary Objective

To determine whether integrating self-efficacy strategies into a multi-joint resistance exercise program results in greater improvements in fat mass compared with multi-joint resistance exercise alone.

5.2 Secondary Objectives

To assess improvements in body composition, psychological outcomes, physical performance (e.g., muscle strength, muscular endurance, balance, and functional mobility), cardiovascular parameters, and metabolic biomarkers associated with cardiometabolic health following the intervention.

6. Study Design

This study was designed as a two-arm, parallel-group, randomized controlled trial conducted over a 12-week intervention period. Participants were stratified by sex, age group (60–69 and 70–75 years), and body mass index (overweight or obese) prior to randomization. A

computer-generated random allocation sequence with a 1:1 allocation ratio was used to assign participants to either the multi-joint resistance exercise combined with self-efficacy strategies (MJS) group or the multi-joint resistance exercise (MJ) group. Allocation concealment was maintained using sequentially numbered, opaque, sealed envelopes prepared by an independent researcher who was not involved in participant recruitment, assessment, or intervention delivery. Outcome assessors and laboratory personnel were blinded to group allocation throughout the study. The study followed the CONSORT 2010 guidelines and was prospectively registered with the Chinese Clinical Trial Registry (ChiCTR2400081386) before participant enrollment.

7. Methodology

Primary and secondary outcomes were assessed at baseline (week 0) and post-intervention (week 12), including self-efficacy in multi-joint exercise, body composition, physical function, and blood biomarkers. Outcome assessments were performed by trained assessors using standardized procedures. Outcome assessors and laboratory personnel were blinded to group allocation.

7.1 Self-Efficacy Assessment

A validated self-efficacy questionnaire with high internal consistency (Cronbach's alpha = 0.90) was used. It comprised four parts: general health data, exercise knowledge, self-efficacy beliefs, and expected outcomes, with adapted scoring criteria based on Bloom's taxonomy.

7.2 Body Composition

Body composition was assessed using a bioelectrical impedance analyzer (Omron HBF-375, Japan). Measurements included body weight, body mass index, percentage body fat, fat mass, fat-free mass, skeletal muscle mass, and visceral fat level. Fat mass was defined as the primary outcome of the study.

7.3 Physical function

A set of physical function measures followed the guidelines from the Senior Fitness Test.

7.3.1 Muscle strength testing was conducted for both the upper and lower body using a back and leg dynamometer, Takei Scientific Instruments, Takei Back and Leg Dynamometer T.K.K.5002, Japan as well as a hand grip dynamometer, Takei Scientific Instruments, Takei Hand Grip Dynamometer T.K.K.5401, Japan) Each test was performed twice, and the best value was recorded.

7.3.2 Muscular endurance was assessed for both upper and lower body using the 30-second arm curl test and the 30-second chair stand test. For the arm curl test, participants used dumbbells weighing 2 kg for men and 1 kg for women. The number of repetitions completed within 30 seconds was recorded for each test.

7.3.3 Balance testing was conducted for both static and dynamic balance using the single-leg stance test and the Timed Up and Go test. All assessments were performed according to standardized procedures by trained assessors.

7.4 Blood Collection and Biochemical Analysis

Venous blood samples were collected by a certified medical technologist from the antecubital vein after an overnight fasting period of at least 8 hours. Blood for glucose and lipid profile analyses was collected into serum separator tubes, while blood for HbA1c analysis was

collected into dipotassium ethylenediaminetetraacetic acid (K_2EDTA) tubes. All biochemical analyses were performed immediately following collection at a certified clinical laboratory within the Faculty of Allied Health Sciences, Chulalongkorn University, Pathumwan, Thailand.

Fasting plasma glucose and lipid profiles (total cholesterol, triglycerides, high-density lipoprotein cholesterol, and low-density lipoprotein cholesterol) were analyzed using an automated chemistry analyzer (Beckman Coulter AU480, USA) employing enzymatic colorimetric methods. The intra-assay coefficient of variation (CV) for glucose and lipid measurements was less than 3%.

Glycated hemoglobin (HbA1c) was measured using high-performance liquid chromatography (HPLC) on a Bio-Rad D-10™ Hemoglobin Testing System (Bio-Rad Laboratories, USA). The intra-assay CV for HbA1c measurement was below 2%.

High-sensitivity C-reactive protein (hs-CRP) was assessed via an immunoturbidimetric assay using the same chemistry analyzer (Beckman Coulter AU480). The intra-assay CV for hs-CRP was less than 5%. All assays were performed according to the manufacturers' protocols and quality control standards. Blood samples were processed and analyzed within two hours of collection to ensure data accuracy.

8. Safety Considerations

All exercise sessions were conducted under the direct supervision of the principal investigator and trained research assistants to ensure participant safety and immediate response to any adverse events. A standardized procedure was established for the identification, documentation, and follow-up of any adverse events or incidents occurring during the intervention period.

Emergency protocols, including access to first aid and contact with emergency medical services, were developed in advance and made available on-site throughout the intervention period. Participants were pre-screened for contraindications to exercise, and appropriate warm-up and cool-down routines were included in every session to minimize the risk of injury or health complications.

No serious adverse events were anticipated based on previous studies using similar exercise protocols in comparable populations; however, all unexpected events were to be documented and reported to the ethics committee as required.

9. Follow-Up

Follow-up assessments were conducted immediately after completion of the 12-week intervention to assess the primary and secondary outcomes. No long-term follow-up was planned after the conclusion of data collection. However, participants were advised to contact the research team if they experienced any delayed adverse effects or concerns related to the intervention. Contact information was provided to all participants, and procedures were in place to document and address any reports submitted after study completion.

10. Data Management and Statistical Analysis

Statistical analyses were performed using IBM SPSS Statistics version 26 (IBM Corp., Armonk, NY, USA). Analyses followed the intention-to-treat principle. Linear mixed-effects models fitted using restricted maximum likelihood (REML) estimation were used to evaluate the

effects of group, time, and group × time interaction. Group, time, and their interaction were treated as fixed effects, with participant included as a random intercept. An autoregressive covariance structure [AR(1)] was specified to account for repeated measurements over time. Estimated marginal means (EMMs), standard errors (SEs), 95% confidence intervals (CIs), and p-values were reported. Pairwise comparisons were adjusted using the Bonferroni correction where appropriate. Statistical significance was defined as $p < 0.05$.

11. Quality Assurance

Quality assurance and quality control procedures were implemented throughout the study to ensure compliance with the protocol, consistency in intervention delivery, and data integrity. All trainers and research staff adhered to a standardized intervention manual, and were trained in protocol-specific procedures prior to study initiation.

The research team conducted weekly internal audits to verify compliance with intervention procedures, monitor session fidelity, and confirm timely and accurate data entry. Data were double-entered and cross-checked to minimize transcription errors.

Although no independent Data and Safety Monitoring Board was established due to the minimal-risk nature of the intervention, adverse events were monitored and reviewed by the principal investigator weekly. The study was conducted in accordance with ethical guidelines and Good Clinical Practice principles. Outcome assessors and laboratory personnel remained blinded to group allocation throughout the study.

12. Expected Outcomes

The study is expected to demonstrate improvements in body composition, psychological outcomes, cardiovascular parameters, physical function, and selected metabolic outcomes among overweight and obese older adults. Greater improvements are expected in the MJS group, particularly for self-efficacy and selected physiological outcomes. Findings related to metabolic biomarkers, particularly HbA1c, will be interpreted cautiously.

13. Dissemination and Publication Policy

The findings of this study will be disseminated through peer-reviewed journals and presentations at national and international academic conferences. The principal investigator will lead the publication process. All contributing members of the research team will be acknowledged appropriately based on their roles. In addition, a summary of the findings will be shared with the participating community center to ensure local impact and understanding. Where applicable, results will be made available to policymakers or public health authorities to inform future community-based interventions.

14. Duration of the Project

The project was implemented in four key phases. Ethical approval was obtained in November 2023. Coordination with the data collection site was conducted between December 2023 and January 2024. Recruitment and intervention were carried out from February to April 2024, followed by post-intervention testing and data analysis from April to October 2024.

15. Problems Anticipated

Potential challenges include participant dropout and scheduling conflicts, particularly among older adults with limited availability. To address this, reminder systems (e.g., phone calls,

line group notifications) were implemented. Flexible scheduling and transport assistance were provided where needed. Risk of adverse events was minimized by supervised training and safety protocols.

16. Project Management

The principal investigator was responsible for overall project coordination, ethical oversight, and final data analysis and reporting. The research team assisted with participant recruitment, data collection, the informed consent process, and the delivery of the intervention according to the standardized protocol.

17. Ethics

The study received ethical approval from the Chulalongkorn University Institutional Review Board (IRB No. 244/66). Written informed consent was obtained from all participants prior to participation. The study adhered to the Declaration of Helsinki and Good Clinical Practice guidelines.

18. Informed Consent Forms

Informed consent forms (ICFs) were developed in Thai to ensure full comprehension by participants. An English version of the ICF is available upon request. The consent process included verbal explanation, time for questions, and voluntary written agreement. Participants were informed of their right to withdraw at any time without penalty.

Part 2: Administrative Information

19. Budget

The project budget included expenses for exercise materials (e.g., dumbbells, sandbags), laboratory testing (e.g., blood glucose, lipid profiles), and participant materials. A detailed, itemized budget is available in the annex.

20. Other Support

No external funding sources supported this project aside from the institutional grants received. The project was solely funded by the 90th Anniversary of Chulalongkorn University, Rachadapisek Sompote Fund and the Faculty of Sports Science Fund, Chulalongkorn University.

21. Collaboration

The project was conducted in collaboration with the Senior Citizens' Quality of Life Development Center, Nonthaburi City Municipality, which supported recruitment, logistics, and provided the venue for intervention sessions.

22. Links to Other Projects

This study is an independent investigation and is not formally linked to any other ongoing or completed research projects.

23. CV of Investigators

Curriculum Vitae of the principal investigator and co-investigators are available upon request.

24. Other Research Activities

The Principal Investigator is not currently engaged in any other research activities that conflict with or compromise the conduct of this study.

25. Financing and Insurance

This study was financially supported by the 90th Anniversary of Chulalongkorn University, Rachadapisek Sompote Fund and the Faculty of Sports Science Fund, Chulalongkorn University. No additional insurance was required, as the study involved minimal risk and all procedures were approved by the Institutional Review Board.
