# Supplementary material for: Effects of integrating self-efficacy strategies into multi-joint resistance exercise for overweight and obese older adults: A randomized controlled trial
Source: PLoS One. 2026 Aug 21;21(8):e0335830. doi: 10.1371/journal.pone.0335830 (PMC13496450; doi:10.1371/journal.pone.0335830)
Supplement: Supplementary file 4 [file pone.0335830.s005.pdf]

Response to the Reviewers

Dear Editor and Reviewers,

We would like to thank the Editor and the Reviewers for their careful evaluation of our manuscript. We greatly appreciate the insightful and constructive comments, which have helped us substantially improve the clarity, methodological rigor, and overall quality of the manuscript.

Response to Additional Editor Comments

We acknowledge the Editor's summary and agree with the overall assessment of the manuscript. In response, we have undertaken a comprehensive major revision to address all identified concerns regarding methodological clarity, statistical rigor, data presentation, and interpretation.

Specifically, we reanalyzed all outcomes using an intention-to-treat framework with linear mixed-effects models, replacing liberal post-hoc testing and reporting estimated marginal means with 95% confidence intervals.

Methodological procedures, including randomization, allocation concealment, assessor roles, power calculation assumptions, attrition handling, and intervention fidelity, have been clarified in detail to improve transparency and reproducibility. Important potential confounders, such as dietary intake, medication use, and physical activity outside the intervention, are now explicitly discussed and acknowledged as limitations, particularly in relation to cardiometabolic outcomes.

In addition, the manuscript has undergone substantial language editing and structural refinement in accordance with PLOS One standards. Tables and figures have been carefully revised to ensure numerical accuracy, consistent units, and appropriate *p*-value formatting. The discussion has been revised to provide a more balanced interpretation of findings, including unexpected or adverse results, and to expand the limitations accordingly.

We believe these revisions substantially strengthen the rigor, clarity, and interpretability of the manuscript and address the Editor's concerns regarding confidence in the study conclusions.

In response to the reviewers' comments, we have undertaken major revisions across multiple sections of the manuscript, including the introduction, methods, results, discussion, and tables. These revisions include a comprehensive reanalysis of the data using an intention-to-treat framework with linear mixed-effects models, clearer methodological reporting, and a more cautious interpretation of the findings. In light of these substantive revisions, we have revised the manuscript title from "Self-efficacy–enhanced multi-joint exercise improves fitness and cardiometabolic health in overweight and obese older adults: A randomized controlled trial" to "Effects of integrating self-efficacy strategies into multi-joint resistance exercise for overweight and obese older adults: A randomized controlled trial" This change was made to ensure that the title accurately reflects the revised analytical approach and the more nuanced interpretation of the findings, emphasizing the integration of self-efficacy strategies rather than implying definitive improvements across outcomes.

Given the extent of the revisions, we have provided a clean revised version of the manuscript rather than a tracked-changes document. All revised or newly added text has been highlighted using a color-coding system to indicate the reviewers addressed: blue for comments from Reviewers 1, yellow for comments from Reviewer 2, green for comments from Reviewer 1 and 2, purple for comments from Reviewers 2 and 3, and gray for comments common to all three reviewers. Detailed explanations of each modification are provided below with

corresponding page and line numbers.Below, we present a point-by-point response to each reviewer comment. Reviewer comments are reproduced in italic font, followed by our responses.

Reviewer 1

Comment 1: The manuscript would benefit from further refinement and improvement in several areas.

Response: We appreciate this comment and agree that refinement was warranted. Accordingly, we have undertaken substantial revisions throughout the manuscript, including restructuring sections, clarifying methods and analyses, and improving the presentation of results and tables. These changes have enhanced the clarity and rigor of the manuscript.

Comment 2: In the abstract, mean SD, 95%CI values are to be presented apart from p value.

Response: We agree with the reviewer and have revised the abstract to improve the clarity and transparency of statistical reporting. Participant characteristics and outcome estimates are now reported using model-based estimates (estimated marginal means ± standard errors [SE]), consistent with the use of linear mixed-effects models in the statistical analysis.

In addition, between-group differences in change are now presented with corresponding 95% confidence intervals (CI), while *p*-values are reported separately, in accordance with the journal's reporting recommendations. The statistical description in the abstract has also been updated to explicitly reflect the use of linear mixed-effects models under the intention-to-treat principle.

These revisions are provided in the revised Abstract (Page 2, Lines 38–42).

Comment 3: Line 90: The exact location is to be stated. e.g. hospital etc.

Response: We have revised the study design subsection in the materials and methods section to clearly specify the exact study location. The manuscript now states that the randomized controlled trial was conducted at the Elderly Quality of Life Development Center, Nonthaburi City Municipality, Thailand.

This revision can be found in the revised Materials and Methods section under the Study design subsection (Page 6, Lines 118-119).

Comment 4: Line 97: The person who performed the randomization and concealment is to be stated.

Response: We have revised the randomization and allocation concealment description in the materials and methods section to clearly specify who performed the randomization and how allocation was concealed. The manuscript now states that randomization was conducted by an independent researcher not involved in participant recruitment or outcome assessment, using a computerized random number generator, and that group allocation was concealed using sealed, opaque envelopes.

These revisions are provided in the revised materials and methods section under the study design subsection (Page 6, Lines 125-131).

Comment 5: Line 107: The type of power analysis used in the G Power software should be specified.

Response: We have revised the Sample size calculation section to explicitly specify the type of power analysis performed using G*Power. The manuscript now states that an a priori power analysis was conducted using an F-test for repeated-measures ANOVA with a within–between interaction, including detailed assumptions for effect size, significance level,

statistical power, number of groups, repeated measurements, and the assumed correlation among repeated measures.

These revisions are provided in the revised materials and methods section under the sample size calculation subsection (Page 6-7, Lines 133–143).

Comment 6: Line 109: Also, to state the sample size for each group.

Response: We have revised the participants subsection in the materials and methods section to clearly report the sample size for each group at recruitment, as well as the final numbers included in the intention-to-treat analysis. The manuscript now specifies that 38 participants were initially randomized (MJS: n = 19; MJ: n = 19), and that following exclusions and dropouts, 34 participants were included in the final analysis (MJS: n = 18; MJ: n = 16), with group-specific demographic characteristics also reported.

These revisions are provided in the revised materials and methods section under the participants subsection (Page 7, Lines 145-152).

Comment 7: Line 101: The name of the guidelines/regulations is to be stated.

Response: We have revised the manuscript to explicitly state the guidelines and regulations followed in conducting the study. The methods section now specifies that all procedures were performed in accordance with the Declaration of Helsinki and the Consolidated Standards of Reporting Trials (CONSORT) guidelines.

These revisions are provided in the revised materials and methods section under the study design subsection (Page 6, Lines 129-131).

Comment 8: Line 110: Usually a certain percentage figure of attrition rates is to be mentioned e.g. 10%, 20% etc to be added to generate the final sample size.

Response: We have revised the manuscript to explicitly report the attrition rate as a percentage. The participants subsection in the materials and methods section now states that four participants withdrew during the intervention period, corresponding to an attrition rate of 11%, and that 34 participants were consequently included in the final analysis.

These revisions are provided in the revised Materials and Methods section under the Participants subsection (Page 7, Lines 145–150).

Comment 9: Line 121: The exclusion criteria is to be stated.

Response: We have revised the participants subsection in the materials and methods section to clearly specify the exclusion criteria. The manuscript now states that participants were excluded if they withdrew consent, experienced illness or injury that prevented continued participation, or attended fewer than 80% of the prescribed exercise sessions.

These revisions are provided in the revised materials and methods section under the participants subsection (Page 8, Lines 159-161).

Comment 10: Line 126, Line 159: The language version of the questionnaire should be stated, as with other questionnaires if not already specified.

Response: We have revised the manuscript to explicitly specify the language versions of the questionnaires used. The Materials and Methods section now states that participants completed the Thai-language versions of the Physical Activity Readiness Questionnaire (PAR-Q+) and the multi-joint exercise–specific self-efficacy questionnaire.

These revisions are provided in the revised materials and methods section, under the Study protocol subsections (Page 8, Lines 165-167).

Comment 11: Line 151: Detailed information about the 12-week program should be provided, including who conducted it and who performed the assessments.

Response: We have revised the Intervention and outcome assessment descriptions in the materials and methods section to provide more detailed information regarding the 12-week program. The manuscript now clearly states that all outcome assessments were conducted jointly by the principal investigator and trained research assistants following standardized procedures.

In addition, we have clarified how intervention fidelity was monitored, including documentation of adherence to the self-efficacy protocol through structured observation, attendance records, and communication via a dedicated LINE application group, as well as monitoring of exercise volume and participation using attendance logs at each supervised session.

These revisions are provided in the revised materials and methods section under the Intervention subsection (Page 8-9, Lines 180-183, Page 9-10, Lines 194-204).

Comment 12: Line 163: The study/reference is to be cited. Details on who performed the measurements of body composition, physical function, muscle strength, muscular endurance, and balance is to be provided.

Response: We have revised the measurement section in the materials and methods to clearly specify both the personnel responsible for conducting each assessment and the relevant supporting references. The manuscript now states that assessments of self-efficacy, body composition, and physical function were performed by trained research assistants under the supervision of the principal investigator, following standardized and validated protocols, while blood chemistry measurements were performed by two certified medical technologists from the Faculty of Allied Health Sciences, Chulalongkorn University.

In addition, appropriate references have been added for all measurement procedures, including the self-efficacy questionnaire, body composition assessment using bioelectrical impedance analysis, and physical function tests covering muscle strength, muscular endurance, balance, and mobility.

These revisions are provided in the revised materials and methods section, under the Measurement subsections (Page 11, Lines 213–218).

Comment 13: Line 218: Typo analyzes

Response: The term "analyzes" has been corrected to "analysis" in the revised manuscript.

These revisions are provided in the revised Materials and Methods section, under the Statistic analysis subsections (Page 14, Lines 274).

Comment 14: Line 238: To include the word mean ± sd Table 2 0.000#, Table 3 0.00. 0.000: Technically the p-value cannot be zero (to use symbol p <)

Response: We would like to clarify that the statistical analysis was revised to use linear mixed-effects models (LMM) rather than repeated-measures ANOVA. Accordingly, outcome values are now reported as estimated marginal means (EMM) ± standard errors (SE) with 95% confidence intervals (CI), which reflect model-based estimates rather than raw descriptive statistics (mean ± SD). In addition, the results tables have been fully updated based on the revised LMM analyses. As a result, no p-values are reported as 0.00 or 0.000 in the revised Tables 2-4. All p-values are now reported appropriately in accordance with standard statistical conventions.

These revisions are provided in the revised Results section (Pages 14–24, Lines 292–390). Corresponding updates have also been made to Tables 2-4 and Figure 2 (Pages 36).

Comment 15: Line 300-301: SD values to be inserted.

Response: We appreciate the reviewer's suggestion regarding the inclusion of SD values. In response to earlier comments, the statistical analysis was revised, and all results are now reported based on linear mixed-effects models (LMM). Accordingly, outcome values are presented as estimated marginal means (EMM) ± standard errors (SE) with 95% confidence intervals (CI), which reflect model-based estimates rather than raw descriptive statistics (mean ± SD). Therefore, SD values were not inserted in this section, as they are not appropriate for LMM-derived estimates. The revised results now consistently report EMM ± SE with 95% CI, and these values have been included for all statistically significant outcomes.

These revisions are provided in the revised Results section (Pages 14–24, Lines 292–390).

Comment 16: Line 318: The presentation of '* p < 0.05 vs. Pre-test, # p < 0.05 vs. MJ group,' is to be improved.

Response: We agree that the previous notation could be improved for clarity. In response, the statistical analysis and presentation of results have been revised. The manuscript no longer uses symbolic comparisons (#) to denote significance. Instead, results are now reported based on linear mixed-effects models and presented as estimated marginal means (EMM) ± standard errors (SE) with 95% confidence intervals (CI). The revised tables and figure footnotes now clarify that change EMM represents within-group changes from pre- to post-intervention, while between-group differences in change represent the contrast of change scores between groups with corresponding 95% CIs. All p-values now correspond to the time × group interaction effects, with statistical significance defined as $p < 0.05$.

These revisions are provided in the revised Results section and updated Tables (Pages 14–24, Lines 292–390).

Comment 17: Line 319: The statement could be improved. e.g Non-normally distributed continuous variables between groups were compared using the Mann–Whitney U test.

Response: We agree that the original statement required clarification. In response, the statistical analysis has been revised, and the manuscript no longer relies on non-parametric group comparisons such as the Mann–Whitney U test. Instead, all continuous outcomes are now analyzed using linear mixed-effects models, which appropriately account for repeated measurements and do not require strict normality assumptions. Accordingly, the Statistical Analysis section has been updated to reflect this revised analytical approach, and the previous statement has been removed.

These revisions are provided in the revised Statistical Analysis and Results sections (Pages 14–24, Lines 292–390).

Comment 18: In the results section, for the presentation of certain p-values, it would be better to use a consistent format, e.g., (mean ± sd vs mean ± sd, p-value), (mean ± sd vs mean ± sd, p-value) and not (mean ± sd vs mean ± sd), (mean ± sd vs mean ± sd) p-value, p-value respectively.

Response: We agree that a consistent format for presenting p-values improves clarity. In response, we have revised both the statistical analysis and the presentation of results. The manuscript no longer reports pairwise comparisons using raw descriptive statistics (mean ± SD). Instead, all results are now presented using estimated marginal means (EMM) ± standard errors (SE) with 95% confidence intervals (CI) derived from linear mixed-effects models. Accordingly, p-values are now reported consistently and correspond exclusively to the time × group interaction effects, and they are described explicitly within the text and tables rather than using mixed or inconsistent formats. The previous presentation formats have been removed.

These revisions are provided in the revised Results section and updated Tables sections (Pages 14–24, Lines 292–390).

Comment 19: The presentation of the effect size is insufficient; the 95% confidence interval should also be included

Response: We agree that reporting effect sizes together with their 95% confidence intervals improves the interpretability of the results. In response, the statistical analysis and presentation have been revised, and 95% confidence intervals are now reported for all effect estimates, including between-group differences in change derived from the linear mixed-effects models.

These revisions are provided in the revised Results section and updated tables (Pages 14–24, Lines 292–390).

Comment 20: Some references do not follow the journal's format or are missing required information.

Response: We acknowledge the importance of accurate and consistent reference formatting. In response, we have carefully reviewed all references and revised them to ensure full compliance with the PLOS One reference style, including the inclusion of all required information such as complete author lists, article titles, journal names, volume and issue numbers, page ranges where applicable, and DOI numbers when available. These revisions have been applied throughout the revised manuscript.

Reviewer 2

Comment 1: An overall recommendation would be "major revision" rather than outright acceptance or rejection, as the study addresses an important question with an appropriate design but needs methodological clarifications, statistical tightening, and substantial language/structural refinement to reach the standard of a high-impact clinical trial report.

Response: Thank you for this thoughtful and constructive overall assessment of our manuscript. We appreciate the reviewer's recognition that the study addresses an important research question using an appropriate design. We fully agree that methodological clarification, strengthened statistical reporting, and substantial language and structural refinement were necessary to improve the quality of the manuscript.

In response, we have undertaken a comprehensive revision of the manuscript. Specifically, we have clarified key methodological aspects, refined the statistical analysis by implementing linear mixed-effects models with appropriate reporting of estimated marginal means and 95% confidence intervals, and substantially revised the language and structure throughout the manuscript to enhance clarity, consistency, and scientific rigor. We believe that these revisions have significantly strengthened the manuscript and aligned it more closely with the standards expected of a high-quality clinical trial report.

We sincerely thank the reviewer for the insightful comments and believe that the revised manuscript addresses all concerns raised.

Comment 2: Standardize "overweight and obese older adults" throughout and correct any "overweight and obesity" phrasing.

Response: We have carefully reviewed the entire manuscript and standardized the terminology throughout. All instances have been revised to consistently use the term

"overweight and obese older adults", and any incorrect or inconsistent phrasing (e.g., "overweight and obesity") has been corrected accordingly.

These revisions have been applied throughout the revised manuscript.

Comment 3: In the abstract, (1) name the primary outcome(s), (2) indicate analytic framework (group × time repeated measures), and (3) add at least one effect size or absolute mean difference for key outcomes (e.g., fat mass, HbA1c).

Response: We have fully revised the abstract to address all aspects of this comment. Specifically, we now clearly define body composition as the primary outcome, with blood biochemistry and physical function evaluated as secondary outcomes. We also explicitly describe the analytic framework using linear mixed-effects models under the intention-to-treat principle, testing group × time interactions, and report absolute between-group differences with 95% confidence intervals for key outcomes (e.g., resting heart rate and HbA1c). We note that the numerical results presented in the revised abstract differ slightly from those in the original submission. These changes reflect the implementation of a more rigorous statistical approach (linear mixed-effects models with estimated marginal means and ITT analysis), as recommended during the revision process. The abstract has been updated accordingly to ensure consistency with the revised analyses and results presented in the manuscript.

These revisions are provided in the revised Abstract section (Pages 2–3, Lines 23-48).

Comment 4: Introduction and literature: Add 1–2 concise paragraphs explicitly summarizing prior combined exercise + psychological/self-efficacy interventions in older adults and state how your trial advances this evidence (population, design, outcomes, integration of SE into each session).

Response: We have revised and expanded the Introduction to explicitly summarize prior studies combining exercise with psychological or self-efficacy–based interventions in

older adults. The revised text now outlines the typical psychological strategies used, their reported effects on adherence, motivation, and selected health outcomes, and key limitations of previous approaches, including the delivery of psychological components as adjunct or separate sessions and the predominant focus on aerobic or mixed exercise modalities.

In addition, we have clearly articulated how the present trial advances the existing evidence by targeting overweight and obese older adults, employing a randomized comparative design, evaluating a broad range of pre-specified physical, metabolic, and psychological outcomes, and, importantly, systematically integrating self-efficacy strategies into each session of a progressive multi-joint resistance training program. The Introduction has been revised accordingly to ensure coherence between prior literature, the identified research gap, and the rationale for the current study.

These revisions are provided in the revised Introduction section (Pages 3-5, Lines 50-115).

Comment 5: Clarify that BMI thresholds follow Asian criteria and explicitly position your work within an Asian older-adult context.

Response: We have clarified the BMI thresholds used in this study and explicitly specified that they follow the criteria recommended for Asian populations. The manuscript has also been revised to clearly position the study within the context of Asian older adults.

These revisions are provided in the revised materials and methods section, under the Participants subsections (Page 7, Lines 153-154).

Comment 6: Methods: Clearly specify primary and secondary outcomes and ensure these align with the power calculation; justify the assumed effect size.

Response: We have now clearly specified the primary and secondary outcomes and ensured their alignment with the sample size calculation. Body composition was defined as the

primary outcome, and fat mass was therefore used as the basis for the a priori power calculation. The assumed effect size (f = 0.40) was derived from a previously published randomized controlled trial examining the effects of resistance exercise on body composition in obese older adults.

In addition, these clarifications have been incorporated consistently throughout the manuscript, including the abstract, the methods section (sample size calculation), and the Outcome measures/Measurements section, to improve transparency, coherence, and methodological consistency.

These revisions are provided in the revised abstract (Page 2, Lines 28-30) and materials and methods section, under the Sample size calculation subsections (Page 6, Lines 133-135) and under the measurement subsections (Page 11, Lines 211-213).

Comment 7: Describe how randomization sequence was generated, how allocation concealment was implemented in practice, and how outcome assessors were blinded (if they were); if blinding was incomplete, state this explicitly.

Response: We have clarified the randomization, allocation concealment, and blinding procedures in the methods section. Randomization was generated using a computerized random number generator by an independent researcher not involved in participant recruitment or outcome assessment, and allocation concealment was implemented using sealed, opaque envelopes. Due to the nature of the exercise-based intervention, blinding of participants, exercise instructors, and outcome assessors was not feasible, and this has now been explicitly stated as a methodological limitation in the manuscript.

These revisions are provided in the revised materials and methods section, under the Study design subsections (Page 6, Lines 125-129).

Comment 8: For the self-efficacy questionnaire, move detailed scoring and level definitions to supplementary material and keep the main text concise (structure, validation sample, reliability indices).

Response: We have revised the manuscript accordingly, as recommended, by moving the detailed scoring procedures and level definitions of the self-efficacy questionnaire to the Supplementary Material. In the main text, we now retain only concise and essential information, including the questionnaire structure, validation sample, and reliability indices, to improve clarity and readability of the Methods section.

These revisions are provided in the revised Materials and Methods section, under the Measurement subsections (Page 11, Lines 219-227).

Comment 9: Explain eligibility threshold for "low to moderate self-efficacy," including cut-off scores.

Response: We have revised the Methods section to clearly explain the eligibility threshold for "low to moderate self-efficacy." Specifically, participants were classified as having low to moderate self-efficacy based on questionnaire scores ranging from 10 to 24, with scores of 10–16 indicating low self-efficacy and 17–24 indicating moderate self-efficacy. This information has now been explicitly included in the eligibility criteria.

These revisions are provided in the revised Materials and Methods section, under the Participants subsections (Page 7-8, Lines 158-159).

Comment 10: Clarify fidelity monitoring: how was adherence to the SE protocol and exercise intensity/volume documented and quantified?

Response: We have revised the Methods section to clarify intervention fidelity monitoring. Adherence to the self-efficacy (SE) protocol was documented through structured observation and session attendance records, with ongoing communication via a dedicated LINE

application group to support participant engagement and reinforce SE strategies. Exercise training fidelity was monitored by recording attendance at each supervised exercise session, and adherence was quantified as the percentage of completed sessions relative to those prescribed. Exercise participation and volume were delivered in accordance with the study protocol, with all sessions continuously supervised by the principal investigator and trained research assistants to ensure protocol adherence. These details have now been explicitly described in the Methods section.

These revisions are provided in the revised Materials and Methods section, under the Intervention subsections (Page 9-10, Lines 194-204).

Comment 11: Specify whether analyses were intention-to-treat or per-protocol and how missing post-test data from dropouts were handled.

Response: We have revised the Statistical analysis section to clearly specify that all analyses were conducted according to the intention-to-treat principle using linear mixed-effects models. This approach was adopted to reduce potential bias associated with participant dropout. Missing post-test data resulting from withdrawals were handled within the linear mixed-effects modeling framework, which allows the inclusion of all available data under the assumption of missing at random without requiring data imputation. These details have now been explicitly stated in the manuscript.

These revisions are provided in the revised materials and methods section, under the Statistical analysis subsections (Page 14, Lines 274-285) and Results section, under the Participant flow and analysis approach subsections (Page 14, Lines 286-290)

Comment 12: Consider using a more conservative post hoc test than LSD or justify the choice and acknowledge increased type I risk.

Response: In response, we have revised the statistical analysis approach and no longer rely on post hoc tests such as the LSD procedure. Instead, changes over time and between-group differences were analyzed using linear mixed-effects models, with fixed effects for group, time, and the group × time interaction, and participants included as a random effect.

This model-based approach allows direct estimation of between-group differences over time without the need for multiple post hoc comparisons, thereby reducing the risk of inflated Type I error. Estimated marginal means with 95% confidence intervals were derived from the models, and statistical significance was assessed using two-tailed tests with $p < 0.05$. These revisions have been implemented throughout the manuscript and are now described in the Statistical analysis section.

Comment 13: Results: Carefully proofread all tables for numeric or typographic inconsistencies (e.g., muscle mass value in the MJ group) and ensure variable labels are accurate and units consistent.

Response: We have carefully proofread all tables to identify and correct any numeric or typographic inconsistencies, including the previously noted muscle mass value in the MJ group. In addition, we have verified that all variable labels are accurate and that measurement units are used consistently across all tables. These revisions have now been implemented throughout the Results section.

These revisions are provided in the revised Results section and updated Tables sections (Pages 14–24, Lines 292–390).

Comment 14: Orient results more clearly around primary outcomes and interaction effects; clearly distinguish within-group changes from between-group differences and avoid overstating within-group significance as evidence of superiority.

Response: We have revised the Results section to more clearly orient the presentation around the primary outcome and group × time interaction effects. Within-group changes and between-group differences are now explicitly distinguished, with between-group differences emphasized where appropriate. We have also revised the wording throughout the Results section to avoid overstating within-group significance as evidence of between-group superiority. These changes were implemented to improve clarity and ensure accurate interpretation of the findings.

These revisions are provided in the revised Results section and updated Tables sections (Pages 14–24, Lines 292–390).

Comment 15: Add 95% confidence intervals for key between-group differences, particularly for fat mass, BMI, and HbA1c.

Response: We have revised the Results section to include 95% confidence intervals for key between-group differences, particularly for fat mass, BMI, and HbA1c. In addition, as the statistical analysis was updated to a linear mixed-effects modeling approach under the intention-to-treat principle, the reporting of results has been comprehensively revised to ensure consistency with the updated analyses. All relevant tables and text have been updated accordingly.

These revisions are provided in the revised Results section and updated Tables sections (Pages 14–24, Lines 292–390).

Comment 16: Discussion and conclusion: Shorten repetitive text on self-efficacy theory and its behavioral consequences; instead, focus on what this specific trial adds empirically.

Response: We have revised both the Discussion and Conclusion sections to reduce repetitive descriptions of self-efficacy theory and its behavioral implications, and to focus more explicitly on the empirical contributions of the present trial. In addition, these sections have been comprehensively updated to ensure consistency with the revised statistical analyses and results. These revisions were made to improve clarity, conciseness, and relevance.

These revisions are provided in the revised Discussion and updated Conclusion section (Pages 24–28, Lines 391–481).

Comment 17: Rephrase speculative explanations (e.g., hs-CRP differences driven by self-efficacy) more cautiously and explicitly label them as hypotheses.

Response: We have revised the Discussion section to rephrase speculative explanations more cautiously and explicitly frame them as hypotheses where appropriate. In addition, the Discussion has been comprehensively updated to ensure consistency with the revised statistical analyses and results following the updated analytical approach. These revisions were made to improve clarity and scientific rigor.

These revisions are provided in the revised Discussion section (Pages 24–27, Lines 391–471).

Comment 18: Expand the limitations to include sample size, single-center Thai setting, BIA limitations, potential unblinding, and absence of dietary control, then temper practical recommendations accordingly.

Response: We have expanded the Limitations section to explicitly address the modest sample size, the single-center study design within a Thai older-adult population, the limitations

of bioelectrical impedance analysis for body composition assessment, the lack of participant blinding due to the nature of the exercise intervention, and the absence of strict dietary control. We have also tempered the practical implications accordingly and highlighted the need for larger, multi-center studies with more comprehensive monitoring in future research. These revisions have been incorporated into the Discussion section.

These revisions are provided in the revised Discussion section (Pages 27, Lines 455–471).

Comment 19: In the conclusion, highlight the main concrete contributions (e.g., feasibility of integrating SE strategies into routine group exercise; added effect on adiposity and HbA1c) and propose specific next steps (larger, multicenter, longer-term trials; examination of cost and implementation).

Response: We have revised the Conclusion section to more clearly highlight the main empirical contributions of the study, including the feasibility of integrating self-efficacy strategies into routine group exercise and their added benefits on selected physiological and metabolic outcomes. We have also expanded the Conclusion to propose specific next steps, including larger, multicenter, and longer-term trials, as well as the evaluation of implementation and cost-related considerations. These revisions have been incorporated accordingly.

These revisions are provided in the revised Conclusion section (Pages 27-28, Lines 472–481).

Comment 20: References and writing: Harmonize reference formatting (journal abbreviations, DOI style, capitalization) in line with PLOS ONE's style guide.

Response: Thank you for this comment. We have revised and harmonized the reference formatting throughout the manuscript to ensure consistency with the PLOS ONE style guide,

including journal abbreviations, DOI formatting, and capitalization. These corrections have now been implemented accordingly.

These revisions are provided in the revised References section (Pages 28-33, Lines 482–596).

Comment 21: Undertake a thorough language and style edit to correct grammar, typos, and formatting artifacts and to shorten long sentences, especially in the Introduction and Discussion

Response: We have thoroughly revised the manuscript to correct grammatical errors, typographical mistakes, and formatting issues, and to improve clarity by shortening and refining long sentences, particularly in the Introduction and Discussion sections. These edits have been implemented throughout the manuscript.

Comment 22: With these revisions, the paper would present a more rigorous, clearly articulated, and practically useful contribution to the literature on behaviorally informed exercise interventions for overweight and obese older adults.

Response: We appreciate your recognition of the potential contribution of this work. We have carefully addressed all comments and revised the manuscript accordingly to improve methodological rigor, clarity of presentation, and practical relevance. We believe that these revisions have substantially strengthened the manuscript and enhanced its contribution to the literature on behaviorally informed exercise interventions for overweight and obese older adults.

Reviewer3:

Comment 1: This manuscript explores the effects of a self-efficacy–enhanced multi-joint exercise program on cardiometabolic and functional outcomes in overweight and obese older adults. Although the study addresses an important public health topic, several significant methodological, statistical, and reporting limitations currently weaken the strength of the conclusions.

Response: Thank you for this thoughtful and constructive overall assessment of our manuscript. We appreciate the recognition of the public health relevance of our research question and the detailed feedback provided. In response, we have undertaken a comprehensive revision of the manuscript, including substantial methodological clarification, reanalysis of the data using a more rigorous statistical framework, and extensive language and structural editing. Our detailed responses to each point are provided below.

Comment 2: Methodological and Technical Concerns: Despite the randomized design, the final sample size after dropout (n = 33) is small and substantially limits the statistical power of the study. The lack of a true non-exercise control group makes it impossible to determine whether observed changes are attributable to the interventions or reflect natural temporal variation. Moreover, the predominance of female participants further restricts generalizability.

Response: We fully acknowledge these methodological limitations. The modest final sample size after dropout, the absence of a non-exercise control group, and the predominance of female participants have now been explicitly addressed in the Limitations section of the Discussion. We have clarified that these factors may limit statistical power and generalizability and warrant cautious interpretation of the findings. These issues are also highlighted as important considerations for future studies employing larger, more diverse samples and the inclusion of non-exercise control groups.

These revisions are provided in the revised Discussion section (Pages 27, Lines 457–473).

Comment 3. Several reported cardiometabolic findings—most notably the increases in fasting glucose, triglycerides, and resting heart rate in the multi-joint exercise group—are physiologically concerning and insufficiently explained. The absence of detailed dietary control, medication tracking, or physical activity monitoring outside the intervention significantly weakens causal interpretation.

Response: We acknowledge that the observed cardiometabolic changes, including increases in fasting glucose, triglycerides, and resting heart rate in the multi-joint exercise group, warrant cautious interpretation. We have now explicitly addressed these concerns in the Limitations section, noting that the absence of detailed dietary control, medication tracking, and monitoring of physical activity outside the intervention limits causal interpretation of these findings. These limitations are highlighted to ensure appropriate contextualization of the results.

These revisions are provided in the revised Discussion section (Pages 27, Lines 455–471).

Comment 4. Statistical Weaknesses: While basic statistical procedures are described, there are notable concerns regarding analytic rigor. The widespread use of the LSD post-hoc test in the presence of numerous outcome variables substantially increases the risk of false-positive findings. No clear intention-to-treat analysis is reported despite participant attrition, which undermines the validity of the randomized design. Furthermore, the reporting of effect sizes for non-parametric tests lacks transparency.

Response: We fully agree with these concerns and have addressed them comprehensively. The statistical analysis has been substantially revised. Specifically, we

replaced repeated-measures ANOVA and liberal post-hoc testing with linear mixed-effects models under the intention-to-treat principle, including fixed effects for group, time, and group × time interaction, and random intercepts for participants. This approach provides a more robust and transparent framework for handling repeated measures and missing data. Effect estimates are now reported as estimated marginal means with standard errors and 95% confidence intervals, and between-group differences in change are consistently presented with confidence intervals. The use of LSD post-hoc testing has been removed.

These revisions are provided in the revised Materials and Methods section, under the Statistical analysis subsections (Page 14, Lines 274-285) and Results section (Pages 14–24, Lines 292–390)

Comment 5. Data Availability: The authors state that data are provided in the manuscript and supporting files, with further release planned through a public repository. Full compliance with PLOS ONE policy must be verified at the time of publication.

Response: We confirm that the Data Availability Statement has been reviewed and updated to comply with PLOS ONE policy. The minimal dataset underlying the findings is provided in the Supporting Information and will be deposited in a public repository upon acceptance, with unrestricted access unless ethical constraints apply. This information is now clearly stated in the revised manuscript.

Comment 6. Language and Presentation Deficiencies: The manuscript suffers from widespread grammatical errors, unclear sentence structures, inconsistent terminology, and formatting problems. These issues significantly impair readability and require comprehensive professional English editing before the manuscript can be considered for publication.

Response: We acknowledge this concern and have undertaken a comprehensive language and formatting revision of the entire manuscript. Sentences have been rewritten for clarity and conciseness, terminology has been standardized, and tables and figures have been carefully checked for consistency and numerical accuracy. The manuscript has undergone thorough English editing to improve readability and professional presentation in line with PLOS ONE standards.

Comment 7. Ethical and Reporting Issues: While ethical approval and informed consent are mentioned, insufficient consideration is given to major confounders such as diet, medications, and behavioral changes outside the intervention. This undermines confidence in the reported cardiometabolic outcomes.

Response: We acknowledge the reviewer's concerns regarding potential confounders, including diet, medication use, and behavioral changes outside the intervention. We recognize that these factors may influence cardiometabolic outcomes and have now explicitly addressed this issue in the Limitations section. The manuscript has been revised to clarify that the absence of detailed control and monitoring of these confounders limits causal interpretation and warrants cautious interpretation of the cardiometabolic findings.

Comment 8. Recommendation: In its current form, the manuscript suffers from substantial methodological, statistical, and language-related limitations that preclude firm conclusions. Major revision is required, including stronger statistical control, clearer explanation of adverse and contradictory findings, improved reporting of confounders, and comprehensive language editing.

Response: We appreciate the reviewer's careful evaluation and acknowledge that substantial revisions were necessary. In response, we have undertaken a comprehensive revision of the manuscript to address the methodological, statistical, and language-related

concerns raised. Specifically, we have strengthened the statistical analyses, clarified the interpretation of adverse and contradictory findings, improved the reporting of key confounders, and performed thorough language and style editing throughout the manuscript. We believe that these revisions have substantially improved the rigor, clarity, and reliability of the study and have strengthened the conclusions accordingly.
