# Supplementary material for: Effects of integrating self-efficacy strategies into multi-joint resistance exercise for overweight and obese older adults: A randomized controlled trial
Source: PLoS One. 2026 Aug 21;21(8):e0335830. doi: 10.1371/journal.pone.0335830 (PMC13496450; doi:10.1371/journal.pone.0335830)
Supplement: Supplementary file 5 [file pone.0335830.s006.pdf]

Responds to reviewers

We sincerely thank the Academic Editor and the reviewers for their careful evaluation of our manuscript and for their constructive comments and valuable suggestions. We have carefully considered all comments and have revised the manuscript accordingly. All changes made in the revised manuscript are highlighted using Track Changes. Our point-by-point responses to each comment are provided below, with page and line numbers referring to the revised manuscript.

Reviewer 1: If there are multiple outcomes, a multiple testing correction is recommended when interpreting p-values separately for each outcome. Alternatively, a Multivariate Linear Mixed-Effects Model can be used to analyze all outcomes jointly, provided that the model assumptions are met.

Answer: Thank you for this valuable suggestion. We agree that multiple comparisons may increase the risk of Type I error when interpreting multiple outcomes separately. Accordingly, we performed Bonferroni-adjusted pairwise comparisons following the linear mixed-effects model analyses. The statistical reporting has been revised accordingly, with Bonferroni-adjusted pairwise comparison results now indicated in the tables and described in the Results section. The Statistical Analysis section in the Materials and Methods has also been revised to specify the use of Bonferroni adjustment for post hoc pairwise comparisons (page 14). The corresponding results have been updated throughout the Results section (pages 16–24, Line 326-332, 342-356, 385-393, 405-416, 448-463, 476-487)

Reviewer 4: 1. HbA1c result: internal inconsistency and concerns about robustness.

The reported between-group difference in HbA1c appears internally inconsistent. The point estimate is shown as −0.4, yet the corresponding 95% CI is (−0.2, 0.0), which does not contain the point estimate. This suggests a reporting error or a mismatch between the analytic output and the displayed summary. In addition, the CI suggests a very small effect (bordering on no difference), whereas the point estimate implies a substantially larger change. Clarification of this discrepancy is essential.

More broadly, a −0.4% reduction in HbA1c over 12 weeks in non-diabetic older adults undergoing resistance training alone is physiologically unusual. Given the small sample size, lack of power calculations for metabolic outcomes, absence of data visualization, and potential baseline variability, the robustness of this finding is uncertain. Please interpret this result with caution and discuss the possibility of chance findings or outlier influence.

Answer: Thank you for your careful review and for identifying this inconsistency. Upon re-examining the statistical output, we confirmed that the discrepancy resulted from a reporting error in the manuscript rather than the statistical analysis itself. Specifically, the reported between-group point estimate and its corresponding 95% confidence interval for HbA1c were inadvertently inconsistent. To avoid confusion and ensure accurate reporting, we have removed the erroneous between-group point estimate and the corresponding 95% confidence interval from the Results and tables. The HbA1c results are now reported consistently based on the linear mixed-effects

model and the Bonferroni-adjusted pairwise comparisons. We have carefully checked the remaining statistical results throughout the manuscript to ensure their accuracy. The revised Results section and Table 4 have been updated accordingly (pages 22–24, Line 448-463, 476-487).

Reviewer 4: 2. Need for visualization of individual-level data.

The manuscript currently presents point estimates and p-values, but the distributions of key outcomes (particularly HbA1c and resting heart rate) are not shown. Scatter plots, boxplots, or individual trajectories would greatly help readers evaluate whether group differences might be driven by outliers or baseline imbalances. Providing these figures is important for transparency and for assessing the validity of the reported effects.

Answer: In response to the reviewer's recommendation, we have added scatter plots with overlaid individual participant data for the two key outcomes, resting heart rate (HR) and glycated hemoglobin (HbA1c), to the revised manuscript. These figures allow readers to visualize the distribution of individual observations, assess potential baseline imbalances, and identify any possible outliers. We believe that the inclusion of these individual-level data visualizations improves the transparency of the results and facilitates a more comprehensive evaluation of the reported findings. The corresponding figure has been added to the revised manuscript as Figure 2A,2B (page 35).

Reviewer 4: 3. Lack of attention-matched or sham control condition

Because the self-efficacy intervention includes guided conversation, interpersonal interaction, and structured encouragement, the control group does not adequately control for non-specific social or attentional effects. These factors alone can influence psychological outcomes. Without an attention-matched (sham) control, the specific contribution of the self-efficacy strategies cannot be isolated. This is a meaningful limitation and should be clearly acknowledged.

Answer: We agree that the absence of an attention-matched control group is an important limitation of the study. Accordingly, we have revised the Discussion to acknowledge that the additional guided discussions, interpersonal interaction, and structured encouragement provided to the MJS group may have introduced non-specific social or attentional effects. We have also noted that future studies should include an attention-matched or sham control group to better isolate the specific effects of the self-efficacy intervention. This limitation has been added to the Discussion section (page 28, Line 608-620).

Reviewer 4: 4. Generalizability is restricted due to inclusion criteria

The study includes only participants with low-to-moderate baseline self-efficacy. This is understandable from an intervention design perspective, but it reduces the generalizability of the findings. Older adults with high self-efficacy may respond differently to resistance training or behavioral strategies. This should be highlighted more explicitly in the Limitations section.

Answer: We agree that restricting the study population to older adults with low to moderate baseline self-efficacy may limit the generalizability of our findings. Accordingly, we have revised the Limitations section to explicitly acknowledge that the findings may not be generalizable to older adults with high baseline self-efficacy, who may respond differently to resistance training

and self-efficacy-enhancing behavioral strategies. We have also noted that future studies should include participants with a broader range of baseline self-efficacy levels to improve the generalizability of the findings. This limitation has been added to the Discussion section (page 28, Line 608-620).

Reviewer 4: 5. Trial registration clarity

Reviewer: The study was conducted in Thailand, but the clinical trial was registered in the Chinese Clinical Trial Registry. Please clarify the rationale for choosing this registry rather than a local or internationally recognized alternative. Transparent reporting of registration is important to ensure consistency with preregistered outcomes.

Answer: Reference: https://www.thaiclinicaltrials.org/. At the time of our trial registration, the Thai Clinical Trials Registry (TCTR) was temporarily unable to accept new trial registrations due to operational changes. According to the official announcement dated 10 November 2023, TCTR gradually suspended its operations from 13 November 2023 due to a lack of financial support. During this period, users were unable to create new accounts or submit new trial records. Although existing registered trials with TCTR identification numbers could still be edited and updated, new trial registrations were no longer accepted. Subsequently, TCTR announced on 25 December 2023 that, effective 1 January 2024, its administration would be transferred from the Medical Research Foundation to the Foundation for Human Research Promotion in Thailand. Further details regarding the operation of the registration system were to be announced later. Although TCTR later resumed ordinary operations on 11 March 2024, the registry was not available for new trial registration during the period when our study needed to be registered. Therefore, in order to proceed with the research in a timely manner and ensure that the trial was registered in an internationally recognized clinical trial registry, we registered the study with the Chinese Clinical Trial Registry instead. This decision was made solely because TCTR was temporarily closed for new registrations at the relevant time, and registration with an alternative recognized registry was necessary to allow the research to proceed appropriately.

Reviewer 4: 6. Adherence and session attendance

It is unclear whether adherence or session attendance was monitored. For exercise-based interventions, especially those involving psychological components, reporting adherence is essential for interpreting whether the observed outcomes reflect the intended intervention dose. If available, please include these metrics; if not, this should be acknowledged as a limitation.

Answer: Participant adherence and session attendance were closely monitored throughout the intervention period. Attendance was recorded at every supervised exercise session to ensure compliance with the intervention protocol. All exercise sessions were supervised by the research team, and participant attendance was checked and documented throughout the study. Participants were required to attend at least 80% of the scheduled exercise sessions to be considered adherent to the intervention protocol. We have clarified this information in the Materials and Methods section (page 8, lines 174–180).

Reviewer 4: 7. Statistical analysis: multiple comparison concerns and model transparency

Although linear mixed-effects modeling is appropriate for repeated measures, several issues require clarification:

No adjustment for multiple comparisons despite numerous secondary outcomes

Lack of model specification details (random effects structure, covariance structure)

No reporting of model diagnostics

No visualization of residuals or assessment of model assumptions

These issues reduce confidence in the reported statistical inferences. Additional detail is recommended.

Answer: We appreciate the reviewer's suggestions regarding the transparency and reporting of the statistical analyses. In response, we have revised the Statistical Analysis section to provide additional details regarding the linear mixed-effects model specification, including the random-effects structure and the first-order autoregressive [AR(1)] covariance structure. We have also addressed the concern regarding multiple comparisons by performing Bonferroni-adjusted pairwise comparisons following the linear mixed-effects model analyses, and the corresponding statistical results have been updated throughout the manuscript. In addition, model assumptions were evaluated by visual inspection of Q–Q plots and residual plots, which indicated no substantial deviations from normality or homoscedasticity. The Statistical Analysis section has been revised accordingly, and the Results section has been updated to reflect these analyses (Materials and Methods, page 14, lines 300–315; Results, pages 16–24, Line 326-332, 342-356, 385-393, 405-416, 448-463, 476-487).

Reviewer 4: 8. Discussion and conclusion: speculative elements

The Discussion generally follows the data; however, several mechanistic explanations (metabolic or physiological pathways) extend beyond what was directly measured. These should be moderated or clearly framed as speculative. The conclusions should also be tempered given the uncertainty around metabolic outcomes.

Answer: We agree that some mechanistic explanations in the original Discussion extended beyond the variables directly measured in this study. Accordingly, we have revised the Discussion to moderate these interpretations and clearly indicate that the proposed metabolic and physiological mechanisms are speculative and should be interpreted with caution. In addition, we have revised the Conclusion to adopt more cautious wording regarding the metabolic outcomes, acknowledging the uncertainty of these findings and emphasizing the need for confirmation in larger, well-powered randomized controlled trials. These revisions have been incorporated into the Discussion and Conclusion sections (pages 25, 27–30, lines 538–540, 573-582, 591-604, 625-665).
