# Supplementary material for: Effects of integrating self-efficacy strategies into multi-joint resistance exercise for overweight and obese older adults: A randomized controlled trial
Source: PLoS One. 2026 Aug 21;21(8):e0335830. doi: 10.1371/journal.pone.0335830 (PMC13496450; doi:10.1371/journal.pone.0335830)
Supplement: Supplementary file 6 [file pone.0335830.s007.pdf]

Response to Reviewers

Comment 1. Consistency of HbA1c Reporting

The reporting of the HbA1c results should be carefully verified for consistency. The Results section states that Bonferroni-adjusted pairwise comparisons showed significant within-group changes in both the MJS and MJ groups, whereas Table 4 does not clearly indicate these comparisons as statistically significant. Please re-examine the Bonferroni-adjusted pairwise comparison output, report the exact adjusted p-values, ensure consistency among the Results, Table 4, and Figure 2B, and revise the table footnote and significance notation accordingly.

Response: Thank you for this important observation. We carefully re-examined the Bonferroni-adjusted pairwise comparison output for HbA1c. Upon verification, we found that the Bonferroni-adjusted pairwise comparisons did not demonstrate statistically significant within-group pre- to post-intervention changes. Accordingly, we revised the manuscript to ensure complete consistency throughout.

Specifically:

- The Results section was revised to state that a significant group × time interaction was observed for HbA1c, whereas Bonferroni-adjusted pairwise comparisons did not demonstrate statistically significant within-group changes (Results, Page 21-23).
- Table 4 and its footnote were revised so that the significance notation accurately reflects the Bonferroni-adjusted results (Table 4, Pages 21-22).
- Figure 2B and its interpretation were checked and confirmed to be fully consistent with the revised Results and Table 4
- The Discussion, Abstract, and Conclusion were also revised to ensure that the interpretation of the HbA1c findings is appropriately cautious and fully consistent throughout the manuscript. (Abstract, Page 2; Discussion, Pages 24–27; Conclusion, Page 27).

Comment 2. Please carefully proofread the manuscript to eliminate the remaining duplicated or repetitive wording and improve the overall readability.

Response: Thank you for this helpful suggestion. We carefully proofread the entire manuscript and revised the text to improve clarity, consistency, and readability. Specifically:

- Duplicated descriptions of the intervention procedures, participant adherence, and warm-up procedures were removed or consolidated. (Materials and Methods, Pages 8–9).

- Repeated discussion regarding the limitations of bioelectrical impedance analysis (BIA) was reduced so that this limitation is presented only once in the Discussion (Discussion and Conclusion, Pages 24-27).
- Repetitive statements regarding future research directions were consolidated into a single concise statement (Conclusion, Pages 27).
- Redundant wording in the Conclusion was revised to provide a clearer and more concise summary of the study findings (Conclusion, Page 27).
- The fragmented sentence identified by the Editor, together with other minor grammatical inconsistencies throughout the manuscript, was corrected during comprehensive proofreading.

These revisions have improved the overall readability and internal consistency of the manuscript.

We appreciate the Editor's careful review, which helped us improve the clarity, consistency, and overall readability of the manuscript.
