# Supplementary material for: Effects of integrating self-efficacy strategies into multi-joint resistance exercise for overweight and obese older adults: A randomized controlled trial
Source: PLoS One. 2026 Aug 21;21(8):e0335830. doi: 10.1371/journal.pone.0335830 (PMC13496450; doi:10.1371/journal.pone.0335830)
Supplement: Peer review history

# Peer review: Decision Letter 0

**DOI:** 10.1371/journal.pone.0335830.r001

**Authors:** Ankita Sharma

19 Dec 2025

Dear Thanomwong Kritpet,

Thank you for submitting your manuscript to PLOS ONE. After careful consideration, we feel that it has merit but does not fully meet PLOS ONE’s publication criteria as it currently stands. Therefore, we invite you to submit a revised version of the manuscript that addresses the points raised during the review process.

==============================

The manuscript addresses a clinically relevant topic and employs a randomized design; however, across all three reviews there is strong convergence on the need for **major revision**  before the work can meet the methodological, statistical, and reporting standards expected for publication.

==============================

Please submit your revised manuscript by Feb 02 2026 11:59PM, If you will need more time than this to complete your revisions, please reply to this message or contact the journal office at plosone@plos.org. When you’re ready to submit your revision, log on to https://www.editorialmanager.com/pone/ and select the 'Submissions Needing Revision' folder to locate your manuscript file.

Please include the following items when submitting your revised manuscript:

- A letter that responds to each point raised by the academic editor and reviewer(s). You should upload this letter as a separate file labeled 'Response to Reviewers'.
- A marked-up copy of your manuscript that highlights changes made to the original version. You should upload this as a separate file labeled 'Revised Manuscript with Track Changes'.
- An unmarked version of your revised paper without tracked changes. You should upload this as a separate file labeled 'Manuscript'.

If you would like to make changes to your financial disclosure, please include your updated statement in your cover letter. Guidelines for resubmitting your figure files are available below the reviewer comments at the end of this letter.

If applicable, we recommend that you deposit your laboratory protocols in protocols.io to enhance the reproducibility of your results. Protocols.io assigns your protocol its own identifier (DOI) so that it can be cited independently in the future. For instructions see: https://journals.plos.org/plosone/s/submission-guidelines#loc-laboratory-protocols. Additionally, PLOS ONE offers an option for publishing peer-reviewed Lab Protocol articles, which describe protocols hosted on protocols.io. Read more information on sharing protocols at https://plos.org/protocols?utm_medium=editorial-email&utm_source=authorletters&utm_campaign=protocols.

We look forward to receiving your revised manuscript.

Kind regards,

Ankita Sharma, PhD

Academic Editor

PLOS One

Journal Requirements:\

When submitting your revision, we need you to address these additional requirements.

1. Please ensure that your manuscript meets PLOS ONE's style requirements, including those for file naming. The PLOS ONE style templates can be found at

https://journals.plos.org/plosone/s/file?id=wjVg/PLOSOne_formatting_sample_main_body.pdf and

https://journals.plos.org/plosone/s/file?id=ba62/PLOSOne_formatting_sample_title_authors_affiliations.pdf.

2. In the online submission form, you indicated that your data will be submitted to a repository upon acceptance.  We strongly recommend all authors deposit their data before acceptance, as the process can be lengthy and hold up publication timelines. Please note that, though access restrictions are acceptable now, your entire minimal  dataset will need to be made freely accessible if your manuscript is accepted for publication. This policy applies to all data except where public deposition would breach compliance with the protocol approved by your research ethics board. If you are unable to adhere to our open data policy, please kindly revise your statement to explain your reasoning and we will seek the editor's input on an exemption.

3. Please be informed that funding information should not appear in the Acknowledgments section or other areas of your manuscript. We will only publish funding information present in the Funding Statement section of the online submission form. Please remove any funding-related text from the manuscript.

4. Please ensure that you refer to Figure 1 in your text as, if accepted, production will need this reference to link the reader to the figure.

5. If the reviewer comments include a recommendation to cite specific previously published works, please review and evaluate these publications to determine whether they are relevant and should be cited. There is no requirement to cite these works unless the editor has indicated otherwise.

Additional Editor Comments:

While the underlying study question is appropriate and potentially valuable, the reviewers consistently identified **incomplete methodological reporting, insufficient statistical rigor, and weaknesses in data presentation and interpretation**  that currently limit confidence in the conclusions. In particular, deficiencies in statistical analysis, including the absence of a clearly stated intention-to-treat approach, reliance on liberal post-hoc testing, incomplete effect size reporting, and lack of confidence intervals, substantially weaken the inferential strength of the findings.

Methodological clarity requires improvement, especially regarding randomization procedures, allocation concealment, outcome assessor roles, power calculation assumptions, attrition handling, and intervention fidelity. Several key details necessary for reproducibility and transparency are either missing or insufficiently described. Additionally, important confounders such as dietary intake, medication use, and non-intervention physical activity were not adequately controlled or discussed, which is particularly problematic for cardiometabolic outcomes.

From a reporting perspective, the manuscript would benefit from substantial language editing and structural refinement. Reviewers noted frequent grammatical and formatting errors, inconsistent terminology, and non-standard statistical presentation, all of which impede readability and professional clarity. Tables and figures require careful correction for numerical accuracy, consistent units, and appropriate p-value formatting.

Despite these limitations, the reviewers agree that the study has the potential to make a meaningful contribution if these concerns are comprehensively addressed. A **thorough major revision**  is therefore warranted, with particular emphasis on:

- Strengthening and transparently reporting the statistical framework
- Clarifying methodological procedures and eligibility criteria
- Improving interpretation of unexpected or adverse findings
- Expanding and balancing the discussion of limitations
- Undertaking comprehensive language and formatting revision in line with PLOS ONE standards

Reviewer 1:

The manuscript would benefit from further refinement and improvement in several areas.

In the abstract, mean sd, 95%CI values are to be presented apart from p value.

Line 90: The exact location is to be stated. e.g. hospital etc

Line 97: The person who performed the randomization and concealment is to be stated.

Line 107: The type of power analysis used in the G Power software should be specified.

Line 109: Also, to state the sample size for each group.

Line 101: The name of the guidelines/regulations is to be stated.

Line 110: Usually a certain percentage figure of attrition rates is to be mentioned e.g. 10%, 20% etc to be added to generate the final sample size.

Line 121: The exclusion criteria is to be stated.

Line 126, Line 159: The language version of the questionnaire should be stated, as with other questionnaires if not already specified.

Line 151: Detailed information about the 12-week program should be provided, including who conducted it and who performed the assessments.

Line 163: The study/reference is to be cited.

Details on who performed the measurements of body composition, physical function, muscle strength, muscular endurance, and balance is to be provided.

Line 218: Typo analyzes

Line 238: To include the word mean ± sd

Table 2 0.000#, Table 3 0.00. 0.000 : Technically the p-value cannot be zero (to use symbol p < )

Line 300-301: SD values to be inserted.

Line 318: The presentation of ‘* p ˂ 0.05 vs. Pre-test, # p ˂ 0.05 vs. MJ group,’ is to be improved.

Line 319: The statement could be improved. e.g Non-normally distributed continuous variables between groups were compared using the Mann–Whitney U test.

In the results section, for the presentation of certain p-values, it would be better to use a consistent format, e.g., (mean ± sd vs mean ± sd, p-value), (mean ± sd vs mean ± sd, p-value) and not (mean ± sd vs mean ± sd), (mean ± sd vs mean ± sd) p-value, p-value respectively.

The presentation of the effect size is insufficient; the 95% confidence interval should also be included.

Some references do not follow the journal’s format or are missing required information.

Reviewer 2:

An overall recommendation would be “major revision” rather than outright acceptance or rejection, as the study addresses an important question with an appropriate design but needs methodological clarifications, statistical tightening, and substantial language/structural refinement to reach the standard of a high-impact clinical trial report.

Specific, actionable suggestions:

Title/abstract

Standardize “overweight and obese older adults” throughout and correct any “overweight and obesity” phrasing.

In the abstract, (1) name the primary outcome(s), (2) indicate analytic framework (group × time repeated measures), and (3) add at least one effect size or absolute mean difference for key outcomes (e.g., fat mass, HbA1c).

Introduction and literature

Add 1–2 concise paragraphs explicitly summarizing prior combined exercise + psychological/self-efficacy interventions in older adults and state how your trial advances this evidence (population, design, outcomes, integration of SE into each session).

Clarify that BMI thresholds follow Asian criteria and explicitly position your work within an Asian older-adult context.

Methods

Clearly specify primary and secondary outcomes and ensure these align with the power calculation; justify the assumed effect size.

Describe how randomization sequence was generated, how allocation concealment was implemented in practice, and how outcome assessors were blinded (if they were); if blinding was incomplete, state this explicitly.

For the self-efficacy questionnaire, move detailed scoring and level definitions to supplementary material and keep the main text concise (structure, validation sample, reliability indices).

Explain eligibility threshold for “low to moderate self-efficacy,” including cut-off scores.

Clarify fidelity monitoring: how was adherence to the SE protocol and exercise intensity/volume documented and quantified?

Specify whether analyses were intention-to-treat or per-protocol and how missing post-test data from dropouts were handled.

Consider using a more conservative post hoc test than LSD or justify the choice and acknowledge increased type I risk.

Results

Carefully proofread all tables for numeric or typographic inconsistencies (e.g., muscle mass value in the MJ group) and ensure variable labels are accurate and units consistent.

Orient results more clearly around primary outcomes and interaction effects; clearly distinguish within-group changes from between-group differences and avoid overstating within-group significance as evidence of superiority.

Add 95% confidence intervals for key between-group differences, particularly for fat mass, BMI, and HbA1c.

Discussion and conclusion

Shorten repetitive text on self-efficacy theory and its behavioral consequences; instead, focus on what this specific trial adds empirically.

Rephrase speculative explanations (e.g., hs-CRP differences driven by self-efficacy) more cautiously and explicitly label them as hypotheses.

Expand the limitations to include sample size, single-center Thai setting, BIA limitations, potential unblinding, and absence of dietary control, then temper practical recommendations accordingly.

In the conclusion, highlight the main concrete contributions (e.g., feasibility of integrating SE strategies into routine group exercise; added effect on adiposity and HbA1c) and propose specific next steps (larger, multicenter, longer-term trials; examination of cost and implementation).

References and writing

Harmonize reference formatting (journal abbreviations, DOI style, capitalization) in line with PLOS ONE’s style guide.

Undertake a thorough language and style edit to correct grammar, typos, and formatting artifacts and to shorten long sentences, especially in the Introduction and Discussion.

With these revisions, the paper would present a more rigorous, clearly articulated, and practically useful contribution to the literature on behaviorally informed exercise interventions for overweight and obese older adults.

Reviewer 3:

Dear Author,

This manuscript explores the effects of a self-efficacy–enhanced multi-joint exercise program on cardiometabolic and functional outcomes in overweight and obese older adults. Although the study addresses an important public health topic, several significant methodological, statistical, and reporting limitations currently weaken the strength of the conclusions.

1. Methodological and Technical Concerns

Despite the randomized design, the final sample size after dropout (n = 33) is small and substantially limits the statistical power of the study. The lack of a true non-exercise control group makes it impossible to determine whether observed changes are attributable to the interventions or reflect natural temporal variation. Moreover, the predominance of female participants further restricts generalizability.

Several reported cardiometabolic findings—most notably the increases in fasting glucose, triglycerides, and resting heart rate in the multi-joint exercise group—are physiologically concerning and insufficiently explained. The absence of detailed dietary control, medication tracking, or physical activity monitoring outside the intervention significantly weakens causal interpretation.

2. Statistical Weaknesses

While basic statistical procedures are described, there are notable concerns regarding analytic rigor. The widespread use of the LSD post-hoc test in the presence of numerous outcome variables substantially increases the risk of false-positive findings. No clear intention-to-treat analysis is reported despite participant attrition, which undermines the validity of the randomized design. Furthermore, the reporting of effect sizes for non-parametric tests lacks transparency.

3. Data Availability

The authors state that data are provided in the manuscript and supporting files, with further release planned through a public repository. Full compliance with PLOS ONE policy must be verified at the time of publication.

4. Language and Presentation Deficiencies

The manuscript suffers from widespread grammatical errors, unclear sentence structures, inconsistent terminology, and formatting problems. These issues significantly impair readability and require comprehensive professional English editing before the manuscript can be considered for publication.

5. Ethical and Reporting Issues

While ethical approval and informed consent are mentioned, insufficient consideration is given to major confounders such as diet, medications, and behavioral changes outside the intervention. This undermines confidence in the reported cardiometabolic outcomes.

Recommendation:

In its current form, the manuscript suffers from substantial methodological, statistical, and language-related limitations that preclude firm conclusions. Major revision is required, including stronger statistical control, clearer explanation of adverse and contradictory findings, improved reporting of confounders, and comprehensive language editing.

[Note: HTML markup is below. Please do not edit.]

Reviewers' comments:

Reviewer's Responses to Questions

**Comments to the Author**

1. Is the manuscript technically sound, and do the data support the conclusions?

Reviewer #1: Partly

Reviewer #2: Partly

Reviewer #3: Partly

**********

2. Has the statistical analysis been performed appropriately and rigorously?

Reviewer #1: No

Reviewer #2: No

Reviewer #3: No

**********

3. Have the authors made all data underlying the findings in their manuscript fully available?

The PLOS Data policy

Reviewer #1: Yes

Reviewer #2: Yes

Reviewer #3: Yes

**********

4. Is the manuscript presented in an intelligible fashion and written in standard English?

Reviewer #1: Yes

Reviewer #2: No

Reviewer #3: No

**********

Reviewer #1: The manuscript would benefit from further refinement and improvement in several areas.

In the abstract, mean sd, 95%CI values are to be presented apart from p value.

Line 90: The exact location is to be stated. e.g. hospital etc

Line 97: The person who performed the randomization and concealment is to be stated.

Line 107: The type of power analysis used in the G Power software should be specified.

Line 109: Also, to state the sample size for each group.

Line 101: The name of the guidelines/regulations is to be stated.

Line 110: Usually a certain percentage figure of attrition rates is to be mentioned e.g. 10%, 20% etc to be added to generate the final sample size.

Line 121: The exclusion criteria is to be stated.

Line 126, Line 159: The language version of the questionnaire should be stated, as with other questionnaires if not already specified.

Line 151: Detailed information about the 12-week program should be provided, including who conducted it and who performed the assessments.

Line 163: The study/reference is to be cited.

Details on who performed the measurements of body composition, physical function, muscle strength, muscular endurance, and balance is to be provided.

Line 218: Typo analyzes

Line 238: To include the word mean ± sd

Table 2 0.000#, Table 3 0.00. 0.000 : Technically the p-value cannot be zero (to use symbol p < )

Line 300-301: SD values to be inserted.

Line 318: The presentation of ‘* p ˂ 0.05 vs. Pre-test, # p ˂ 0.05 vs. MJ group,’ is to be improved.

Line 319: The statement could be improved. e.g Non-normally distributed continuous variables between groups were compared using the Mann–Whitney U test.

In the results section, for the presentation of certain p-values, it would be better to use a consistent format, e.g., (mean ± sd vs mean ± sd, p-value), (mean ± sd vs mean ± sd, p-value) and not (mean ± sd vs mean ± sd), (mean ± sd vs mean ± sd) p-value, p-value respectively.

The presentation of the effect size is insufficient; the 95% confidence interval should also be included.

Some references do not follow the journal’s format or are missing required information.

Reviewer #2: An overall recommendation would be “major revision” rather than outright acceptance or rejection, as the study addresses an important question with an appropriate design but needs methodological clarifications, statistical tightening, and substantial language/structural refinement to reach the standard of a high-impact clinical trial report.

Specific, actionable suggestions:

Title/abstract

Standardize “overweight and obese older adults” throughout and correct any “overweight and obesity” phrasing.

In the abstract, (1) name the primary outcome(s), (2) indicate analytic framework (group × time repeated measures), and (3) add at least one effect size or absolute mean difference for key outcomes (e.g., fat mass, HbA1c).

Introduction and literature

Add 1–2 concise paragraphs explicitly summarizing prior combined exercise + psychological/self-efficacy interventions in older adults and state how your trial advances this evidence (population, design, outcomes, integration of SE into each session).

Clarify that BMI thresholds follow Asian criteria and explicitly position your work within an Asian older-adult context.

Methods

Clearly specify primary and secondary outcomes and ensure these align with the power calculation; justify the assumed effect size.

Describe how randomization sequence was generated, how allocation concealment was implemented in practice, and how outcome assessors were blinded (if they were); if blinding was incomplete, state this explicitly.

For the self-efficacy questionnaire, move detailed scoring and level definitions to supplementary material and keep the main text concise (structure, validation sample, reliability indices).

Explain eligibility threshold for “low to moderate self-efficacy,” including cut-off scores.

Clarify fidelity monitoring: how was adherence to the SE protocol and exercise intensity/volume documented and quantified?

Specify whether analyses were intention-to-treat or per-protocol and how missing post-test data from dropouts were handled.

Consider using a more conservative post hoc test than LSD or justify the choice and acknowledge increased type I risk.

Results

Carefully proofread all tables for numeric or typographic inconsistencies (e.g., muscle mass value in the MJ group) and ensure variable labels are accurate and units consistent.

Orient results more clearly around primary outcomes and interaction effects; clearly distinguish within-group changes from between-group differences and avoid overstating within-group significance as evidence of superiority.

Add 95% confidence intervals for key between-group differences, particularly for fat mass, BMI, and HbA1c.

Discussion and conclusion

Shorten repetitive text on self-efficacy theory and its behavioral consequences; instead, focus on what this specific trial adds empirically.

Rephrase speculative explanations (e.g., hs-CRP differences driven by self-efficacy) more cautiously and explicitly label them as hypotheses.

Expand the limitations to include sample size, single-center Thai setting, BIA limitations, potential unblinding, and absence of dietary control, then temper practical recommendations accordingly.

In the conclusion, highlight the main concrete contributions (e.g., feasibility of integrating SE strategies into routine group exercise; added effect on adiposity and HbA1c) and propose specific next steps (larger, multicenter, longer-term trials; examination of cost and implementation).

References and writing

Harmonize reference formatting (journal abbreviations, DOI style, capitalization) in line with PLOS ONE’s style guide.

Undertake a thorough language and style edit to correct grammar, typos, and formatting artifacts and to shorten long sentences, especially in the Introduction and Discussion.

With these revisions, the paper would present a more rigorous, clearly articulated, and practically useful contribution to the literature on behaviorally informed exercise interventions for overweight and obese older adults.

Reviewer #3: Dear Author,

This manuscript explores the effects of a self-efficacy–enhanced multi-joint exercise program on cardiometabolic and functional outcomes in overweight and obese older adults. Although the study addresses an important public health topic, several significant methodological, statistical, and reporting limitations currently weaken the strength of the conclusions.

1. Methodological and Technical Concerns

Despite the randomized design, the final sample size after dropout (n = 33) is small and substantially limits the statistical power of the study. The lack of a true non-exercise control group makes it impossible to determine whether observed changes are attributable to the interventions or reflect natural temporal variation. Moreover, the predominance of female participants further restricts generalizability.

Several reported cardiometabolic findings—most notably the increases in fasting glucose, triglycerides, and resting heart rate in the multi-joint exercise group—are physiologically concerning and insufficiently explained. The absence of detailed dietary control, medication tracking, or physical activity monitoring outside the intervention significantly weakens causal interpretation.

2. Statistical Weaknesses

While basic statistical procedures are described, there are notable concerns regarding analytic rigor. The widespread use of the LSD post-hoc test in the presence of numerous outcome variables substantially increases the risk of false-positive findings. No clear intention-to-treat analysis is reported despite participant attrition, which undermines the validity of the randomized design. Furthermore, the reporting of effect sizes for non-parametric tests lacks transparency.

3. Data Availability

The authors state that data are provided in the manuscript and supporting files, with further release planned through a public repository. Full compliance with PLOS ONE policy must be verified at the time of publication.

4. Language and Presentation Deficiencies

The manuscript suffers from widespread grammatical errors, unclear sentence structures, inconsistent terminology, and formatting problems. These issues significantly impair readability and require comprehensive professional English editing before the manuscript can be considered for publication.

5. Ethical and Reporting Issues

While ethical approval and informed consent are mentioned, insufficient consideration is given to major confounders such as diet, medications, and behavioral changes outside the intervention. This undermines confidence in the reported cardiometabolic outcomes.

Recommendation:

In its current form, the manuscript suffers from substantial methodological, statistical, and language-related limitations that preclude firm conclusions. Major revision is required, including stronger statistical control, clearer explanation of adverse and contradictory findings, improved reporting of confounders, and comprehensive language editing.

**********

what does this mean?). If published, this will include your full peer review and any attached files.

If you choose “no”, your identity will remain anonymous but your review may still be made public.

**Do you want your identity to be public for this peer review?**  For information about this choice, including consent withdrawal, please see our Privacy Policy

Reviewer #1: No

Reviewer #2: **Yes:** Yaser Alikhajeh

Reviewer #3: No

**********

[NOTE: If reviewer comments were submitted as an attachment file, they will be attached to this email and accessible via the submission site. Please log into your account, locate the manuscript record, and check for the action link "View Attachments". If this link does not appear, there are no attachment files.]

To ensure your figures meet our technical requirements, please review our figure guidelines: https://journals.plos.org/plosone/s/figures

You may also use PLOS’s free figure tool, NAAS, to help you prepare publication quality figures: https://journals.plos.org/plosone/s/figures#loc-tools-for-figure-preparation.

NAAS will assess whether your figures meet our technical requirements by comparing each figure against our figure specifications.

# Author comment: Author response to Decision Letter 1

**DOI:** 10.1371/journal.pone.0335830.r002

3 Feb 2026

We sincerely thank the editor and all reviewers for their constructive and insightful comments.

All comments have been carefully addressed and responded to in detail in the accompanying Response to Reviewers document.

Corresponding revisions have been made throughout the manuscript, including substantial methodological clarification, reanalysis using an intention-to-treat linear mixed-effects modeling approach, and revision of the manuscript title to reflect the updated analytical framework.

Attachment Submitted filename: Response to Reviewers.pdf

# Peer review: Decision Letter 1

**DOI:** 10.1371/journal.pone.0335830.r003

**Authors:** Mohamed Said

14 Jun 2026

Dear Dr. Kanlayawut,

Thank you for submitting your manuscript to PLOS ONE. After careful consideration, we feel that it has merit but does not fully meet PLOS ONE’s publication criteria as it currently stands. Therefore, we invite you to submit a revised version of the manuscript that addresses the points raised during the review process.

Please submit your revised manuscript within %DATE_REVISION-DUE% from the date of this report. If you will need more time than this to complete your revisions, please reply to this message or contact the journal office at plosone@plos.org. When you're ready to submit your revision, log on to https://www.editorialmanager.com/pone/ and select the 'Submissions Needing Revision' folder to locate your manuscript file.

- A letter that responds to each point raised by the academic editor and reviewer(s). You should upload this letter as a separate file labeled 'Response to Reviewers'.
- A marked-up copy of your manuscript that highlights changes made to the original version. You should upload this as a separate file labeled 'Revised Manuscript with Track Changes'.
- An unmarked version of your revised paper without tracked changes. You should upload this as a separate file labeled 'Manuscript'.

If you would like to make changes to your financial disclosure, please include your updated statement in your cover letter. Guidelines for resubmitting your figure files are available below the reviewer comments at the end of this letter.

If applicable, we recommend that you deposit your laboratory protocols in protocols.io to enhance the reproducibility of your results. Protocols.io assigns your protocol its own identifier (DOI) so that it can be cited independently in the future. For instructions see: https://journals.plos.org/plosone/s/submission-guidelines#loc-laboratory-protocols. Additionally, PLOS ONE offers an option for publishing peer-reviewed Lab Protocol articles, which describe protocols hosted on protocols.io. Read more information on sharing protocols at https://plos.org/protocols?utm_medium=editorial-email&utm_source=authorletters&utm_campaign=protocols.

As the corresponding author, your ORCID iD is verified in the submission system and will appear in the published article. PLOS supports the use of ORCID, and we encourage all coauthors to register for an ORCID iD and use it as well. Please encourage your coauthors to verify their ORCID iD within the submission system before final acceptance, as unverified ORCID iDs will not appear in the published article. *Only*  the individual author can complete the verification step; PLOS staff *cannot*  verify ORCID iDs on behalf of authors.

We look forward to receiving your revised manuscript.

Kind regards,

Mohamed Ahmed Said, Ph.D.

Academic Editor

PLOS One

Journal Requirements:

If the reviewer comments include a recommendation to cite specific previously published works, please review and evaluate these publications to determine whether they are relevant and should be cited. There is no requirement to cite these works unless the editor has indicated otherwise.

[Note: HTML markup is below. Please do not edit.]

Reviewers' comments:

Reviewer's Responses to Questions

**Comments to the Author**

Reviewer #1: (No Response)

Reviewer #4: (No Response)

**********

2. Is the manuscript technically sound, and do the data support the conclusions?

Reviewer #1: Partly

Reviewer #4: Partly

**********

3. Has the statistical analysis been performed appropriately and rigorously?

Reviewer #1: No

Reviewer #4: No

**********

4. Have the authors made all data underlying the findings in their manuscript fully available?

The PLOS Data policy

Reviewer #1: Yes

Reviewer #4: No

**********

5. Is the manuscript presented in an intelligible fashion and written in standard English?

Reviewer #1: Yes

Reviewer #4: Yes

**********

Reviewer #1: The authors have addressed most of the comments.

Minor comment:

If there are multiple outcomes, a multiple testing correction is recommended when interpreting p-values separately for each outcome. Alternatively, a Multivariate Linear Mixed-Effects Model can be used to analyze all outcomes jointly, provided that the model assumptions are met.

Reviewer #4: Thank you for the opportunity to review this revised manuscript. The study addresses an important topic, and the authors present a well‑structured exercise intervention with meaningful psychological outcomes. The manuscript is generally clear and understandable. At the same time, several methodological and interpretational issues require clarification and revision before the manuscript can be considered technically sound according to PLOS ONE criteria.

Below I provide detailed comments that I hope will help strengthen the work.

1. HbA1c result: internal inconsistency and concerns about robustness

The reported between‑group difference in HbA1c appears internally inconsistent. The point estimate is shown as −0.4, yet the corresponding 95% CI is (−0.2, 0.0), which does not contain the point estimate. This suggests a reporting error or a mismatch between the analytic output and the displayed summary. In addition, the CI suggests a very small effect (bordering on no difference), whereas the point estimate implies a substantially larger change. Clarification of this discrepancy is essential.

More broadly, a −0.4% reduction in HbA1c over 12 weeks in non‑diabetic older adults undergoing resistance training alone is physiologically unusual. Given the small sample size, lack of power calculations for metabolic outcomes, absence of data visualization, and potential baseline variability, the robustness of this finding is uncertain. Please interpret this result with caution and discuss the possibility of chance findings or outlier influence.

2. Need for visualization of individual‑level data

The manuscript currently presents point estimates and p‑values, but the distributions of key outcomes (particularly HbA1c and resting heart rate) are not shown. Scatter plots, boxplots, or individual trajectories would greatly help readers evaluate whether group differences might be driven by outliers or baseline imbalances. Providing these figures is important for transparency and for assessing the validity of the reported effects.

3. Lack of attention‑matched or sham control condition

Because the self‑efficacy intervention includes guided conversation, interpersonal interaction, and structured encouragement, the control group does not adequately control for non‑specific social or attentional effects. These factors alone can influence psychological outcomes. Without an attention‑matched (sham) control, the specific contribution of the self‑efficacy strategies cannot be isolated. This is a meaningful limitation and should be clearly acknowledged.

4. Generalizability is restricted due to inclusion criteria

The study includes only participants with low‑to‑moderate baseline self‑efficacy. This is understandable from an intervention design perspective, but it reduces the generalizability of the findings. Older adults with high self‑efficacy may respond differently to resistance training or behavioral strategies. This should be highlighted more explicitly in the Limitations section.

5. Trial registration clarity

The study was conducted in Thailand, but the clinical trial was registered in the Chinese Clinical Trial Registry. Please clarify the rationale for choosing this registry rather than a local or internationally recognized alternative. Transparent reporting of registration is important to ensure consistency with preregistered outcomes.

6. Adherence and session attendance

It is unclear whether adherence or session attendance was monitored. For exercise‑based interventions, especially those involving psychological components, reporting adherence is essential for interpreting whether the observed outcomes reflect the intended intervention dose. If available, please include these metrics; if not, this should be acknowledged as a limitation.

7. Statistical analysis: multiple comparison concerns and model transparency

Although linear mixed‑effects modeling is appropriate for repeated measures, several issues require clarification:

No adjustment for multiple comparisons despite numerous secondary outcomes

Lack of model specification details (random effects structure, covariance structure)

No reporting of model diagnostics

No visualization of residuals or assessment of model assumptions

These issues reduce confidence in the reported statistical inferences. Additional detail is recommended.

8. Discussion and conclusion: speculative elements

The Discussion generally follows the data; however, several mechanistic explanations (metabolic or physiological pathways) extend beyond what was directly measured. These should be moderated or clearly framed as speculative. The conclusions should also be tempered given the uncertainty around metabolic outcomes.

Overall assessment

The study addresses a relevant question and provides valuable psychological outcome data. However, substantial clarification and additional analyses are needed for the physiological outcomes, particularly HbA1c and resting heart rate. Addressing the points above would considerably strengthen the rigor and transparency of the manuscript.

I hope these comments are helpful in further improving the manuscript.

**********

what does this mean?). If published, this will include your full peer review and any attached files.

If you choose “no”, your identity will remain anonymous but your review may still be made public.

**Do you want your identity to be public for this peer review?**  For information about this choice, including consent withdrawal, please see our Privacy Policy

Reviewer #1: No

Reviewer #4: No

**********

[NOTE: If reviewer comments were submitted as an attachment file, they will be attached to this email and accessible via the submission site. Please log into your account, locate the manuscript record, and check for the action link "View Attachments". If this link does not appear, there are no attachment files.]

To ensure your figures meet our technical requirements, please review our figure guidelines: https://journals.plos.org/plosone/s/figures

You may also use PLOS’s free figure tool, NAAS, to help you prepare publication quality figures: https://journals.plos.org/plosone/s/figures#loc-tools-for-figure-preparation.

NAAS will assess whether your figures meet our technical requirements by comparing each figure against our figure specifications.

# Author comment: Author response to Decision Letter 2

**DOI:** 10.1371/journal.pone.0335830.r004

8 Jul 2026

Dear Editorial Office,

We have addressed all of the requested technical issues as requested. Please find the revised submission attached.

Thank you for your consideration.

Attachment Submitted filename: Response_to_Reviewers_auresp_2.pdf

# Peer review: Decision Letter 2

**DOI:** 10.1371/journal.pone.0335830.r005

**Authors:** Mohamed Said

13 Jul 2026

Dear Dr.  Kritpet,

Thank you for submitting your manuscript to PLOS ONE. After careful consideration, we feel that it has merit but does not fully meet PLOS ONE’s publication criteria as it currently stands. Therefore, we invite you to submit a revised version of the manuscript that addresses the points raised during the review process.

Please submit your revised manuscript by Aug 27 2026 11:59PM. If you will need more time than this to complete your revisions, please reply to this message or contact the journal office at plosone@plos.org. When you're ready to submit your revision, log on to https://www.editorialmanager.com/pone/ and select the 'Submissions Needing Revision' folder to locate your manuscript file.

- A letter that responds to each point raised by the academic editor and reviewer(s). You should upload this letter as a separate file labeled 'Response to Reviewers'.
- A marked-up copy of your manuscript that highlights changes made to the original version. You should upload this as a separate file labeled 'Revised Manuscript with Track Changes'.
- An unmarked version of your revised paper without tracked changes. You should upload this as a separate file labeled 'Manuscript'.

If you would like to make changes to your financial disclosure, please include your updated statement in your cover letter. Guidelines for resubmitting your figure files are available below the reviewer comments at the end of this letter.

If applicable, we recommend that you deposit your laboratory protocols in protocols.io to enhance the reproducibility of your results. Protocols.io assigns your protocol its own identifier (DOI) so that it can be cited independently in the future. For instructions see: https://journals.plos.org/plosone/s/submission-guidelines#loc-laboratory-protocols. Additionally, PLOS ONE offers an option for publishing peer-reviewed Lab Protocol articles, which describe protocols hosted on protocols.io. Read more information on sharing protocols at https://plos.org/protocols?utm_medium=editorial-email&utm_source=authorletters&utm_campaign=protocols.

As the corresponding author, your ORCID iD is verified in the submission system and will appear in the published article. PLOS supports the use of ORCID, and we encourage all coauthors to register for an ORCID iD and use it as well. Please encourage your coauthors to verify their ORCID iD within the submission system before final acceptance, as unverified ORCID iDs will not appear in the published article. *Only*  the individual author can complete the verification step; PLOS staff *cannot*  verify ORCID iDs on behalf of authors.

We look forward to receiving your revised manuscript.

Kind regards,

Mohamed Ahmed Said, Ph.D.

Academic Editor

PLOS One

Journal Requirements:

1. If the reviewer comments include a recommendation to cite specific previously published works, please review and evaluate these publications to determine whether they are relevant and should be cited. There is no requirement to cite these works unless the editor has indicated otherwise.

2. Please review your reference list to ensure that it is complete and correct. If you have cited papers that have been retracted, please include the rationale for doing so in the manuscript text, or remove these references and replace them with relevant current references. Any changes to the reference list should be mentioned in the rebuttal letter that accompanies your revised manuscript. If you need to cite a retracted article, indicate the article’s retracted status in the References list and also include a citation and full reference for the retraction notice.

Additional Editor Comments:

I would like to thank the authors for their careful revision of the manuscript and their comprehensive responses to the reviewers' comments. The manuscript has improved substantially in terms of scientific rigor, clarity, and overall presentation. The revised version addresses the major concerns raised during the previous review rounds, and the study is now close to meeting the publication standards of PLOS ONE.

Before the manuscript can be accepted, however, a few minor issues should be addressed to ensure the accuracy and consistency of the statistical reporting and to further improve the quality of the presentation.

Comment 1. Consistency of HbA1c Reporting

The reporting of the HbA1c results should be carefully verified for consistency. The Results section states that Bonferroni-adjusted pairwise comparisons showed significant within-group changes in both the MJS and MJ groups, whereas Table 4 does not clearly indicate these comparisons as statistically significant. To avoid potential confusion, please:

1. Re-examine the Bonferroni-adjusted pairwise comparison output for HbA1c.

2. Report the exact adjusted p-values for the within-group comparisons instead of reporting only "p < 0.05".

3. Ensure complete consistency between the Results text, Table 4, and Figure 2B.

4. Revise the table footnote and significance notation accordingly. If no within-group comparisons remain statistically significant after Bonferroni adjustment, the text and table should be revised to reflect the correct findings.

Comment 2. Minor Editorial Revision

Please carefully proofread the manuscript to eliminate the remaining duplicated or repetitive wording and improve the overall readability. In particular, attention should be given to:

• duplicated descriptions of the intervention and warm-up procedures;

• repeated discussion of the limitations of bioelectrical impedance analysis (BIA);

• repetitive statements regarding future research directions;

• redundant wording in the Conclusion; and

• the fragmented sentence identified on Page 80, Line 523, together with any similar grammatical inconsistencies elsewhere in the manuscript.

Once these minor issues have been satisfactorily addressed, I believe the manuscript will be suitable for publication in PLOS ONE.

[Note: HTML markup is below. Please do not edit.]

Reviewer's Responses to Questions

**Comments to the Author**

Reviewer #1: All comments have been addressed

**********

2. Is the manuscript technically sound, and do the data support the conclusions?

Reviewer #1: (No Response)

**********

3. Has the statistical analysis been performed appropriately and rigorously?

Reviewer #1: (No Response)

**********

4. Have the authors made all data underlying the findings in their manuscript fully available?

The PLOS Data policy

Reviewer #1: (No Response)

**********

5. Is the manuscript presented in an intelligible fashion and written in standard English?

Reviewer #1: Yes

**********

Reviewer #1: (No Response)

**********

what does this mean?). If published, this will include your full peer review and any attached files.

If you choose “no”, your identity will remain anonymous but your review may still be made public.

**Do you want your identity to be public for this peer review?**  For information about this choice, including consent withdrawal, please see our Privacy Policy

Reviewer #1: No

**********

[NOTE: If reviewer comments were submitted as an attachment file, they will be attached to this email and accessible via the submission site. Please log into your account, locate the manuscript record, and check for the action link "View Attachments". If this link does not appear, there are no attachment files.]

To ensure your figures meet our technical requirements, please review our figure guidelines: https://journals.plos.org/plosone/s/figures

You may also use PLOS’s free figure tool, NAAS, to help you prepare publication quality figures: https://journals.plos.org/plosone/s/figures#loc-tools-for-figure-preparation.

NAAS will assess whether your figures meet our technical requirements by comparing each figure against our figure specifications.

# Author comment: Author response to Decision Letter 3

**DOI:** 10.1371/journal.pone.0335830.r006

29 Jul 2026

Dear Academic Editor,

Thank you for the opportunity to revise our manuscript. We have carefully addressed all reviewer and editor comments. Please see the attached Response to Reviewers document for our detailed point-by-point responses.

Thank you for your consideration.

Attachment Submitted filename: Response_to_Reviewers_auresp_3.pdf

# Editor report: Decision Letter 3

**DOI:** 10.1371/journal.pone.0335830.r007

**Authors:** Mohamed Said

5 Aug 2026

Effects of  integrating self-efficacy strategies into multi-joint resistance exercise for overweight and obese older adults: A randomized controlled trial.

PONE-D-25-54625R3

Dear Dr. Thanomwong Kritpet,

We’re pleased to inform you that your manuscript has been judged scientifically suitable for publication and will be formally accepted for publication once it meets all outstanding technical requirements.

Within one week, you’ll receive an e-mail detailing the required amendments. When these have been addressed, you’ll receive a formal acceptance letter and your manuscript will be scheduled for publication.

An invoice will be generated when your article is formally accepted. Please note, if your institution has a publishing partnership with PLOS and your article meets the relevant criteria, all or part of your publication costs will be covered. Please make sure your user information is up-to-date by logging into Editorial Manager at Editorial Manager® and clicking the ‘Update My Information' link at the top of the page. For questions related to billing, please contact billing support.

If your institution or institutions have a press office, please notify them about your upcoming paper to help maximize its impact. If they’ll be preparing press materials, please inform our press team as soon as possible -- no later than 48 hours after receiving the formal acceptance. Your manuscript will remain under strict press embargo until 2 pm Eastern Time on the date of publication. For more information, please contact onepress@plos.org.

Kind regards,

Mohamed Ahmed Said, Ph.D.

Academic Editor

PLOS One

Additional Editor Comments (optional):

Reviewers' comments:

# Editor report: Acceptance letter

**DOI:** 10.1371/journal.pone.0335830.r008

**Authors:** Mohamed Said

PONE-D-25-54625R3

PLOS One

Dear Dr. Kritpet,

I'm pleased to inform you that your manuscript has been deemed suitable for publication in PLOS One. Congratulations! Your manuscript is now being handed over to our production team.

At this stage, our production department will prepare your paper for publication. This includes ensuring the following:

* All references, tables, and figures are properly cited

* All relevant supporting information is included in the manuscript submission,

* There are no issues that prevent the paper from being properly typeset

You will receive further instructions from the production team, including instructions on how to review your proof when it is ready. Please keep in mind that we are working through a large volume of accepted articles, so please give us a few days to review your paper and let you know the next and final steps.

Lastly, if your institution or institutions have a press office, please let them know about your upcoming paper now to help maximize its impact. If they'll be preparing press materials, please inform our press team within the next 48 hours. Your manuscript will remain under strict press embargo until 2 pm Eastern Time on the date of publication. For more information, please contact onepress@plos.org.

You will receive an invoice from PLOS for your publication fee after your manuscript has reached the completed accept phase. If you receive an email requesting payment before acceptance or for any other service, this may be a phishing scheme. Learn how to identify phishing emails and protect your accounts at https://explore.plos.org/phishing.

If we can help with anything else, please email us at customercare@plos.org.

Thank you for submitting your work to PLOS One and supporting open access.

Kind regards,

PLOS ONE Editorial Office Staff

on behalf of

Dr. Mohamed Ahmed Said

Academic Editor

PLOS One
